# A revision of the *Miliusa* (Annonaceae) from China

**DOI:** 10.3897/phytokeys.273.174592

**Published:** 2026-04-21

**Authors:** Zhiyuan Shi, Xueliang Hou

**Affiliations:** 1 School of Environment and Ecology, Xiamen University, Xiamen, 361102, Fujian, China School of Environment and Ecology, Xiamen University Xiamen China; 2 School of Life Sciences, Xiamen University, Xiamen, 361102, Fujian, China School of Life Sciences, Xiamen University Xiamen China

**Keywords:** Malmeoideae, Miliuseae, molecular systematics, new species, new synonyms, taxonomic revision

## Abstract

Based on extensive fieldwork, herbarium and literature studies, and molecular phylogenetic analyses, we present a comprehensive taxonomic revision of the genus *Miliusa* in China. Ten species are recognized, with the following major taxonomic changes: *Miliusa tenuistipitata* is reduced to synonymy with *M. dioeca*; two new species, *M. aurilaveoides* X.L.Hou & Z.Y.Shi, **sp. nov**. and *M. xiaoboi* X.L.Hou & Z.Y.Shi, **sp. nov**., are described; *M. sinensis* is reinstated as distinct from *M. balansae*; and the treatment of *M. cuneata* and *M. velutina* in the “Flora of China” is revised. An identification key and photographs of living plants are provided.

## Introduction

Annonaceae Juss. is the largest family in the order Magnoliales, comprising over 80% of the species in the order and including 108 genera and approximately 2,500 species distributed throughout tropical and subtropical regions. As an early-diverging lineage of angiosperms, it serves as an important model for studies on the evolution and diversification of this family (Nge et al. 2024). Annonaceae is divided into four subfamilies: Anaxagoreoideae, Ambavioideae, Annonoideae, and Malmeoideae (Chatrou et al. 2012; Nge et al. 2024). The genus *Miliusa* Lesch. ex A. DC. belongs to the tribe Miliuseae within the subfamily Malmeoideae and currently comprises 61 species (Mols and Kessler 2003b; Nge et al. 2024).

The genus *Miliusa* was established by Candolle (1832) based on *M. indica* Lesch. ex A. DC. (Turner 2018). Hooker and Thomson (1855) undertook the first taxonomic revision of *Miliusa* and reduced *Hyalostemma* to synonymy under it. Subsequently, the genus *Saccopetalum* was also subsumed into *Miliusa* (Baillon 1867; Pierre 1881; Finet and Gagnepain 1907; Sinclair 1955). Jessup (1988) revised *Miliusa* in Australia. Mols and Kessler (2003a) conducted a taxonomic revision of Austro-Malesian *Miliusa* and proposed that most species occur in mainland Asia, with the center of diversity likely in Thailand or India. Chaowasku and Kessler (2013a, 2013b) recognized four informal groups within *Miliusa*: the *M. mollis* group, the *M. horsfieldii* group (including species formerly placed in *Saccopetalum* Benn.), the *M. velutina* group, and the *M. campanulata* group. Notably, these morphology-based groups do not entirely correspond to the four clades identified through molecular phylogenetic analyses: clade B corresponds to the *M. campanulata* group and clade C to the *M. mollis* group, whereas clades A and D each include species from both the *M. horsfieldii* and *M. velutina* groups. To date, a total of 132 species/varieties have been published in the genus *Miliusa*, comprising 68 accepted species and 2 accepted varieties (WFO Plantlist 2026); among them, 40 species/varieties have been published since the year 2000.

*Miliusa sinensis* Finet & Gagnep. was first recorded for the Chinese flora by Finet and Gagnepain (1907). Subsequent studies have described several new species from China, including *Miliusa glochidioides* Hand.-Mazz., *M. tenuistipitata* W. T. Wang (now *M. dioeca* (Roxb.) Chaowasku & Kessler), *M. chunii* W. T. Wang (as *M. filipes* Merr. & Chun, nom. illeg., non *M. filipes* Ridl.), *M. bannaensis* X. L. Hou (now *M. thorelii* Finet & Gagnep.), and *M. longicarpa* Z. Y. Shi & X. L. Hou (Handel-Mazzetti 1933; Merrill and Chun 1935; Wu and Wang 1957; Hou et al. 2004; Shi and Hou 2024). In addition, one new combination, *Miliusa prolifica* (Chun & F. C. How) P. T. Li (now *M. horsfieldii* (Benn.) Pierre) (Li 1993), and four newly recorded species—*M. cuneata* Craib, *M. velutina* J. D. Hooker & Thomson, *M. dioeca*, and *M. chantaburiana* Damthongdee & Chaowasku—have been documented for China (Yuan 1991; Li and Gilbert 2011; Ding et al. 2023). Notably, *Miliusa velutina* was subsequently excluded from the Chinese flora by Xue and Tan (2016), and the occurrence of *M. chantaburiana* in China is regarded as doubtful (see Discussion). Despite these substantial taxonomic changes, no comprehensive revision of the genus in China has yet been undertaken, and this study aims to address this gap to establish a foundation for future research.

## Materials and methods

### Morphology

Building on many years of field observations of *Miliusa* species indigenous to China, we conducted a comprehensive study of 794 herbarium specimens (1,335 sheets) of the genus *Miliusa* from China (see Suppl. material 1). The specimens examined are primarily deposited in herbaria in China, including AU, FJSI, GFS, GXMG, GXMI, GZAC, GZTM, HGAS, HITBC, IBK, IBSC, JXU, KUN, NAS, NF, PE, QNUN, SN, SYS, SZG, WUK, etc. In addition, we examined further herbarium specimens of *Miliusa*, with particular emphasis on type material, deposited in the herbaria A, B, BM, C, E, F, HM, HN, K, L, MO, NY, P, UC, and US.

Morphological analyses were based on observations of living plants, herbarium specimens, and digital images of specimens. Vegetative and reproductive structures were observed and measured to document morphological variation. All measurements were taken from dried material. The abbreviation “ca.” precedes measurements when precise quantification or counting was impractical or when only a single observation was available. Unless otherwise indicated, all photographs of dried specimens and living plants were taken and processed by the authors.

### Phylogenetics analyses

Based on extensive field investigations, we collected 10 specimens representing seven Chinese *Miliusa* species (excluding *M. chantaburiana* and *M. dioeca*) and three unidentified taxa. Fresh young leaves were dried in silica gel for DNA preservation, and all voucher specimens were deposited in the Xiamen University Herbarium (AU). To meet the objectives of this study, 38 additional *Miliusa* samples from previous studies (Chaowasku and Kessler 2013a; Gan et al. 2022; Damthongdee et al. 2023; Jin et al. 2024) were incorporated, resulting in a total of 40 *Miliusa* species included. The dataset also comprised 24 samples from 24 other genera within the tribe Miliuseae (Damthongdee et al. 2023). *Dendrokingstonia gardneri* Chaowasku (Dendrokingstonieae) and *Monocarpia maingayi* (Hook. f. & Thomson) I. M. Turner (Monocarpieae), both belonging to the subfamily Malmeoideae, were used as outgroup taxa. Phylogenetic trees were constructed using six chloroplast gene markers: *matK* exon, *ndhF* exon, *psbA–trnH* spacer, *rbcL* exon, *trnL–F* spacer, and *ycf1* exon, following Damthongdee et al. (2023). The final dataset comprised 77 Annonaceae samples. A complete taxon list, including species information, voucher details, and sequence database accession numbers, is provided in Suppl. material 2.

We obtained the six chloroplast markers using two complementary approaches. For Sanger sequencing (Method 1), target regions were PCR-amplified following Chaowasku and Kessler (2013a) and sequenced on an ABI 3730xl DNA Analyzer (Sangon Biotech, Shanghai). Sequences were assembled in PhyDE ver. 1 (Müller et al. 2010). For genome mining (Method 2), total genomic DNA was extracted using a modified CTAB protocol (Doyle and Doyle 1987), assessed for integrity via 1% agarose gel electrophoresis, and quantified with a Qubit ver. 2.0 fluorometer (Life Technologies). Approximately 1.5 µg of DNA per sample was sonicated to ~350 bp fragments, library-prepared with the NEBNext Ultra DNA Library Prep Kit (NEB), and sequenced on an Illumina NovaSeq 6000 platform (2 × 150 bp paired-end reads, ca. 2 GB per sample). Chloroplast genomes were assembled *de novo* with NOVOPlasty ver. 2.6.5 (Dierckxsens et al. 2017) using *Miliusa glochidioides* (NC062046) as reference, annotated in PGA (Qu et al. 2019), manually curated in Geneious ver. 9.1.4 (Kearse et al. 2012), and mined for target gene markers.

Sequences were assembled and edited in PhyloSuite (Zhang et al. 2020), aligned with MAFFT (Katoh and Standley 2013), and concatenated into a partitioned supermatrix. Nucleotide substitution models for each partition were selected with ModelFinder (Kalyaanamoorthy et al. 2017) for both maximum likelihood (ML) and Bayesian inference (BI) analyses. ML analyses were performed in IQ-TREE ver. 1.6.8 (Nguyen et al. 2015) with 5,000 ultrafast bootstrap replicates. BI analyses were conducted in MrBayes ver. 3.2.6 (Ronquist et al. 2012), running four Markov chain Monte Carlo (MCMC) chains for 10 million generations with sampling every 100 generations; the first 25% were discarded as burn-in, and consensus trees with posterior probabilities were computed from the remaining samples.

## Results and discussion

### Phylogenetic relationships

The cpDNA dataset based on *matK*, *ndhF*, *psbA–trnH*, *rbcL*, *trnL–F*, and *ycf1* comprises 7,144 base pairs (bp), including 520 parsimony-informative sites. ModelFinder identified the best-fit model for IQ-TREE as K3Pu+F+R3 and for MrBayes as GTR+F+I+G4. The MrBayes analysis yielded an average standard deviation of split frequencies of 0.00341, with all effective sample size (ESS) values after burn-in exceeding 7,200, indicating satisfactory convergence of the runs.

**Figure 1.**
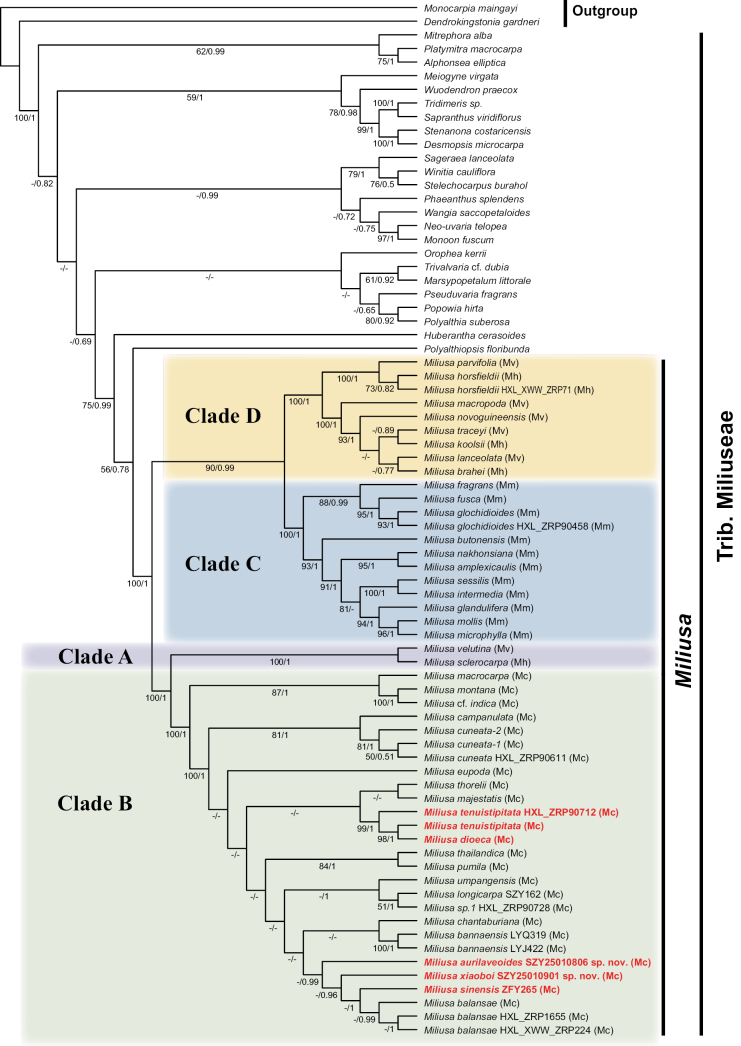
The phylogenetic tree of *Miliusa* was generated using the maximum likelihood (ML) algorithm. Support values are indicated as follows: BS = maximum likelihood bootstrap values, PP = Bayesian posterior probabilities. A dash (–) indicates BS < 50 and PP < 0.5. Mc = *Miliusa campanulata* group; Mh = *M. horsfieldii* group; Mm = *M. mollis* group; Mv = *M. velutina* group; all groups sensu Chaowasku and Kessler (2013a, 2013b).

The tree topologies obtained from ML and BI analyses were largely congruent, particularly within the *Miliusa* clade (Suppl. materials 3, 4). The ML tree is presented in Fig. 1, with bootstrap support (BS) and Bayesian posterior probabilities (PP) indicated at the nodes. *Miliusa* was strongly supported as monophyletic (BS = 100, PP = 1.00) and was recovered as sister to *Polyalthiopsis* with moderate support (BS = 56, PP = 0.78). Within *Miliusa*, the four proposed clades (Fig. 1: clades A–D) received strong support (BS ≥ 90, PP ≥ 0.99). Clade A is sister to clade B, clade C is sister to clade D, and the clade comprising A and B is sister to the clade comprising C and D.

In clade C, *Miliusa fusca* Pierre (Pierre 1881) from Thailand clustered with two *M. glochidioides* samples from China with strong support (BS = 95, PP = 1.00). In clade B, *M. dioeca* (India) was nested among two *M. tenuistipitata* samples from China, forming a strongly supported clade (BS ≥ 98, PP = 1.00). Two newly described species, *Miliusa aurilaveoides* X.L.Hou & Z.Y.Shi, sp. nov. and *M. xiaoboi* X.L.Hou & Z.Y.Shi, sp. nov., are placed in clade B. Although internal relationships within this clade remain partially unresolved, the relevant nodes are well supported (PP ≥ 0.96), with these taxa forming a clade together with *Miliusa sinensis* and *M. balansae* Finet & Gagnep. Different population samples of *Miliusa balansae* cluster together (PP ≥ 0.99), *M. sinensis* is sister to *M. balansae* (PP = 1.00), and *M. xiaoboi* is sister to the clade comprising *M. sinensis* and *M. balansae* (PP = 0.96). *Miliusa aurilaveoides* occupies a basal position in this subclade and is sister to the clade formed by *M. xiaoboi*, *M. sinensis*, and *M. balansae* (PP = 0.99).

Consistent with Chaowasku and Kessler (2013a), our results indicate that phylogenetic relationships within *Miliusa* remain incompletely resolved. While clade B receives strong posterior-probability support (PP ≥ 0.96), its internal topology is poorly resolved. Two Chinese samples of *Miliusa bannaensis* did not cluster with *M. thorelii* from Thailand, differing from Chaowasku’s (2013) proposal to synonymize these species; this discrepancy may reflect the limited resolution of plastid markers or potential misidentification of the *M. thorelii* material. Additionally, *Miliusa* sp. 1 cited by Ding et al. (2023) reported *M. chantaburiana* as a new record for China based on their collections. However, our analysis shows that these Chinese specimens form a distinct clade separate from Thai *Miliusa chantaburiana*, suggesting they may not be conspecific.

### Geographical distribution

The genus *Miliusa* is widely distributed across the Indian subcontinent, southern China, mainland Southeast Asia, the Southeast Asian islands, New Guinea (including the D’Entrecasteaux Islands and Louisiade Archipelago), and northern Australia (Chaowasku and Kessler 2006, 2013a).

In China, the northern distribution limit of *Miliusa* extends from Hunan Province (Jiangyong County) through Guangxi Zhuang Autonomous Region (Guanyang County, Lingchuan County, and Rong’an County); • Guizhou Province (Libo County, Duyun City, Xiuwen County, Chishui City, Anshun City, and Xingyi City); and Yunnan Province (Shizong County, Wenshan City, Gejiu City, Yuanjiang City, Zhenyuan County, Changning County, and Yingjiang County) to Xizang Autonomous Region (Motuo County). The eastern limit runs from Hunan Province (Jiangyong County) through Guangxi Zhuang Autonomous Region (Hezhou City) and Guangdong Province (Xinxing City, Yangchun City) to Hainan Province (Wenchang City). The southern and western limits coincide with the national borders of China.

### Morphology

Based on morphological examination of ten *Miliusa* species from China, we identified novel evidence supporting the monophyly of the *M. mollis* group. According to the taxonomic framework established by Chaowasku and Kessler (2006, 2013a, 2013b), the Chinese *Miliusa* can be divided into three groups: the *M. mollis* group (*M. glochidioides*); the *M. campanulata* group (*M. aurilaveoides* sp. nov., *M. balansae*, *M. cuneata*, *M. longicarpa*, *M. xiaoboi* sp. nov., *M. dioeca* (including *M. tenuistipitata*), *M. thorelii*, and *M. sinensis*); and the *M. horsfieldii* group (*M. horsfieldii*). Distinct differences were observed between *Miliusa glochidioides* and the other species studied.

Firstly, leaf-base asymmetry is a diagnostic trait: among the ten taxa, only *Miliusa glochidioides* consistently exhibits markedly oblique leaf bases, whereas the remaining species display either symmetrical (*M. aurilaveoides*, *M. cuneata*, *M. longicarpa*, and *M. xiaoboi*) or only slightly to moderately oblique bases (*M. balansae*, *M. horsfieldii*, *M. tenuistipitata*, *M. thorelii*, and *M. sinensis*).

Secondly, *Miliusa glochidioides* is characterized by remarkably short petioles, 1.0–1.5 mm in length. The petiole lengths of the other nine species range from 2.0–4.0 mm, with the exception of *Miliusa thorelii*, whose petioles are 5.0–8.0 mm long.

Thirdly, ecological specialization to limestone karst habitats is evident: *Miliusa glochidioides*, *M. aurilaveoides*, and *M. xiaoboi* are endemic to limestone mountainous regions, whereas the remaining seven species exhibit broader ecological amplitudes. Notably, *Miliusa aurilaveoides* and *M. xiaoboi*, as newly described taxa, require further investigation to confirm strict habitat fidelity to limestone substrates.

The combination of extremely short petioles (0–1.5 mm), asymmetrical leaf bases, and association with limestone habitats is also documented in other members of the *Miliusa mollis* group, including *M. fusca*, *M. mollis* Pierre (Pierre 1881), *M. amplexicaulis* Ridl. (Ridley 1910), *M. fragrans* Chaowasku & Kessler, *M. intermedia* Chaowasku & Kessler, *M. nakhonsiana* Chaowasku & Kessler, *M. sessilis* Chaowasku & Kessler (Chaowasku 2013), and *M. ninhbinhensis* Chaowasku & Kessler (Chaowasku 2014). These shared traits provide substantial support for recognizing the *Miliusa mollis* group as a monophyletic lineage within *Miliusa*.

### Taxonomic treatment of the Chinese species

#### Miliusa

Taxon classificationPlantaeMagnolialesAnnonaceae

Lesch. ex A. DC. in Mém. Soc. Phys. Genève 5: 213 (1832)

0674F073-AA02-5FD8-B63E-9DC4BA4240D2

Miliusa Lesch. ex A. DC. in Mém. Soc. Phys. Genève 5: 213 (1832); Hook. f. & Thomson, Fl. Ind. 147 (1855); Fl. Brit. India 1: 86 (1872); Kurz, Forest Fl. Burma 1: 46 (1877); Finet & Gagnep. in Lecomte, Fl. Indo-Chine 1: 109 (1907); J. Sinclair, Gard. Bull. Singapore 14: 377 (1955); Y. Tsiang & P. T. Li, Fl. Reipubl. Popularis Sin. 30(2): 39 (1979); N. T. Ban, Fl. Vietnam 1: 305 (2000); Mols & Kessler, Blumea 48: 421 (2003); P. T. Li & M. G. Gilbert in C. Y. Wu et al., Fl. China 19: 679 (2011); Fl. Thailand 16(1): 167 (2022). Type: Miliusa indica Lesch. ex A. DC. (holotype: G! [G00402293]; isotypes: P! [P00432374, P00432375]).
Hyalostemma Wall., Numer. List 6434 (1832). Type: Hyalostemma roxburghiana Wall., nom. illegit, superfl. (≡ Miliusa dioeca (Roxb.) Chaowasku).
Saccopetalum Benn., Pl. Jav. Rar. 165, t. 35 (1840). Type: Saccopetalum horsfieldii Benn.

##### Description.

Shrubs, small to medium-sized trees, rarely large trees, 2–40(–55) m tall, evergreen, rarely deciduous or semi-deciduous. Annual branches predominantly pubescent to varying degrees, sparsely pubescent to tomentose, becoming glabrescent with age; older branches mostly glabrous, with or without sparse lenticels, longitudinally wrinkled. Leaves chartaceous to subcoriaceous, rarely membranaceous, elliptic, ovate-elliptic, obovate-elliptic or oblong-elliptic, rarely ovate, obovate, lanceolate or oblong, 1.8–36 cm long (mostly 5–13 cm), 1–18 cm wide (mostly 2–5 cm), length:width ratio 1.8–3.3; base obtuse-rounded to cuneate, rarely cordate or subcordate, usually asymmetrical; apex mostly acuminate or acute, rarely rounded or mucronate; midvein adaxially flat, slightly impressed or raised, abaxially prominently raised; lateral veins 4–22 per side (mostly 6–15), straight to arching, anastomosing near the margin; tertiary venation reticulate; adaxial leaf surface glabrous, pubescent, or pubescent only along the midvein; abaxial surface usually hairy; petioles 0–7(–15) mm long, adaxially grooved or flat, abaxially wrinkled, rarely thickened at the base. Inflorescences simple cymes with 1–10 flowers, axillary, subaxillary, or rarely terminal; inflorescence rachis very short or absent; peduncle present, 0.5–2 mm long, rarely up to 20 mm long, thickened during fruiting; pedicels 0.4–33 cm long (mostly 1–3 cm), 0.5–1 mm in diam., thickened to 0.7–2(–3.5) mm during fruiting; bracts and bracteoles usually present, 1–2(–4) of each, triangular-ovate, 1–3 × 1–2 mm, pubescent. Flowers bisexual, occasionally unisexual. Calyx valvate, sepals free, rarely slightly connate at base, ovate, triangular to broadly ovate-triangular, rarely lanceolate or linguiform, 0.5–3(–10) mm long, 0.5–2(–3.5) mm wide, length:width ratio 0.7–3, persistent in fruit; outer petals valvate, free, ovate, triangular or narrowly ovate-triangular, slightly longer than sepals, 1–4(–13) mm long, 1–3(–6) mm wide, length:width ratio 1.5–5, not persistent in fruit; inner petals yellow, red, purplish red or pale yellowish white, ovate, ovate-triangular or ovate-elliptic, 2.3–42 mm long, 2–15 mm wide, appressed or free from base to middle, saccate or nearly flat, with glands and coloured spots, colourless translucent “window-like” regions, or with a coloured patch along the midvein. Androecium of the “miliusoides” type; stamens 3–232, usually 20–70, with 1–6 sterile stamens occasionally present. Anthers 2-locular, ellipsoid, 0.5–1.3(–1.8) mm long, 0.5–1(–1.3) mm wide, with a longitudinal groove; connective curved, apex obtuse or rarely acute, slightly exceeding the anther; filaments conspicuous, 0.2–0.5(–0.9) mm long. Carpels 3–80, usually 10–40; ovaries ellipsoid, (0.5–)1–2(–2.5) mm long, 0.5–1 mm in diam., glabrous or pubescent; ovules lateral, mostly 1–2 per carpel, rarely 4–12, in 1 or 2 rows; style absent; stigmas ellipsoid, cylindrical, capitate or subglobose, 0.3–2 mm long, 0.3–0.5 mm in diam., glabrous or pubescent. Torus pubescent, conical, subglobose or hemispherical, 1.5–2.5 mm long, 2–3 mm in diam., enlarging to subglobose in fruit. Fruiting pedicel 0.4–33 cm long (mostly 1–7 cm), 0.7–2 mm in diam.; fruit with 1–40 monocarps; monocarps generally spherical, subglobose to ellipsoid, base asymmetric, apex rounded or mucronate, base rounded or tapering to the enlarged pedicel apex, 6–19 mm long, 6–14 mm wide; immature fruits green, maturing red, purplish red, purplish black or black, berry-like, usually smooth, with minute tubercles when dry; pericarp thin, ca. 0.5 mm thick; stipe terete, occasionally slightly quadrangular, 0–50(–65) mm long. Seeds 1–10 per monocarp, subglobose or ellipsoid (flattened on 1–2 faces when several), 6–9 × 4–8 mm or 11–17 × 7–15 mm; endosperm ruminate, with lamellate ruminations divided into four parts.

##### Phenology.

Most species flower from February to May, rarely from September to December, with a few species flowering in multiple seasons. Fruits typically mature 1–5 months after flowering.

##### Distribution.

Tropical and subtropical regions of Asia to Oceania, including Australia, Bangladesh, Bhutan, Cambodia, China, India, Indonesia, Laos, Malaysia, Myanmar, Nepal, Papua New Guinea, the Nicobar and Andaman Islands, the Philippines, Singapore, Sri Lanka, Thailand, and Vietnam.

##### Habitat.

Tropical and subtropical lowland regions, mainly in humid and warm conditions; numerous species also occur at higher elevations (up to 2000 m) or in seasonally dry limestone karst. Most species are primarily associated with evergreen broad-leaved forests, while some inhabit deciduous or semi-evergreen forests.

### Key to the Species of *Miliusa* in China

**Table:** 

1	Inner petals ca. 4 mm long and with a transverse gland at the base on the inner surface, not saccate at base; petiole 1–1.5 mm long	**1. *Miliusa glochidioides***
–	Inner petals 15–40 mm long, with or without longitudinal glandular structures along the midrib on the inner surface, saccate at base; petiole 2–8 mm long	**2**
2	Inner petals fully open at anthesis, apex slightly reflexed; ovules 4–10 per ovary; large deciduous trees, up to 25 m tall	**2. *Miliusa horsfieldii***
–	Inner petals appressed from base to middle, only the upper parts free at anthesis, apex distinctly reflexed; ovules 1–3 per ovary; evergreen small trees or shrubs, 2–5(12) m tall	**3**
3	Petiole 5–8 mm long; inflorescences 2–6(–9)-flowered; pedicels ca. 10 mm long; leaf blades 6.5–10 cm wide	**10. *Miliusa thorelii***
–	Petiole 2–4 mm long; inflorescences 1(–3)-flowered; pedicels 10–83 mm long; leaf blades 2–6.5 cm wide	**4**
4	Sepals 4–7 mm long; flowers bisexual, sometimes unisexual	**8. *Miliusa dioeca***
–	Sepals 1.5–3 mm long; flowers bisexual	**5**
5	Flower buds pyriform, distinctly constricted at the middle, resembling a bulb syringe; outer petals red, broadly ovate, 4–5 mm long	**3. *Miliusa aurilaveoides***
–	Flower buds conical, not constricted at the middle; outer petals green, rarely red, ovate or narrowly ovate, 2–4 mm long	**6**
6	Flowers red at maturity; pedicels and fruiting pedicels longer than 35 mm; stipes of fruiting carpels 11–30 mm long	**7**
–	Flowers yellow at maturity; pedicels 15–30 mm long, fruiting pedicels 20–35 mm long; stipes of fruiting carpels 8–12 mm long	**9**
7	Monocarps apex not apiculate; monocarp stipes 11–16 mm long; fruiting pedicels 8–12 cm long	**4. *Miliusa longicarpa***
–	Monocarps apex apiculate; monocarp stipes 15–30 mm long; fruiting pedicels 5–9 cm long	**8**
8	Outer petals ovate, 2.5 × 1.5 mm; sepals broadly ovate, 1.5–2 × 1.5–2 mm; leaves abaxially usually glabrous or sparsely pubescent, hairs 0.1–0.2 mm long	**5. *Miliusa balansae***
–	Outer petals narrowly ovate, 3–4 × 1 mm; sepals ovate, 2–3 × 1 mm; leaves abaxially usually densely pubescent, hairs ca. 0.5 mm long	**6. *Miliusa sinensis***
9	Outer petals broadly ovate, 1.5 × 1.2 mm, not reflexed at anthesis, inner petals with conspicuous red to purplish-red patches adaxially; leaves lateral veins 9–12 pairs	**7. *Miliusa xiaoboi***
–	Outer petals narrowly ovate-triangular, 3–4 × 1–1.5 mm, reflexed at anthesis; inner petals with inconspicuous pale red patches adaxially; leaves lateral veins 14–18 pairs	**9. *Miliusa cuneata***

#### 1. Miliusa glochidioides

Taxon classificationPlantaeMagnolialesAnnonaceae

Hand.-Mazz., Sinensia 3: 185 (1933)

C01BF2BE-7DE3-5766-A361-E3D8E1CEA059

Fig. 2

Miliusa glochidioides Hand.-Mazz., Sinensia 3: 185 (1933); P. T. Li & M. G. Gilbert in Fl. China 19: 680 (2011). Type: China. Guangxi (Kwangsi): Tan-Ngar, 10 Li E of Hoo-Chi (Hechi City), alt. 567 m, 12 Jul 1928, R. C. Ching 6403 (lectotype: NAS! [NAS00070768], designated by Turner (2018); isolectotypes: IBSC! [IBSC0079201, IBSC0003361], NAS! [NAS00321712], NY! [NY00026113], PE! [PE00028366]).
Orophea anceps auct. non Pierre, H. L. Li in Journ. Arn. Arb. 24 (4): 445 (1943); P. T. Li in Acta Phytotax. Sin. (1976), pro parte; Y. Tsiang et P. T. Li in Fl. Reip. Popul. Sin. 30 (2): 37 (1979), pro parte; S. H. Yuan in Fl. Yunnan. 5: 14 (1991), pro parte; P. T. Li in Fl. Guangxi 1: 120 (1991), pro parte.

##### Chinese name.

广西野独活 Guǎng Xī Yě Dú Huó.

**Figure 2.**
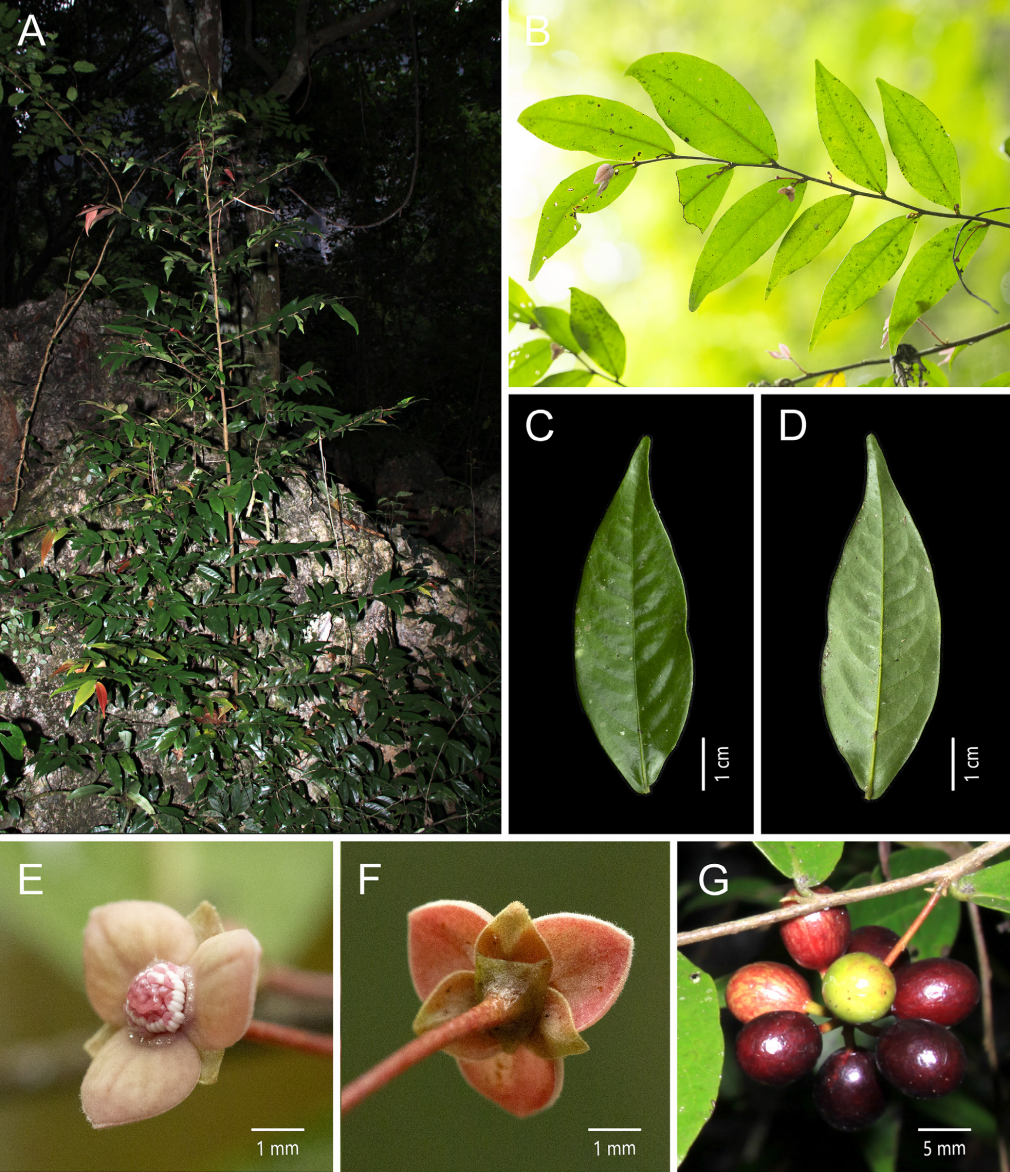
*Miliusa glochidioides* Handel-Mazzetti. **A**. Plant; **B**. Flowering branch; **C**. Adaxial view of the leaf blade; **D**. Abaxial view of the leaf blade; **E**. Bottom view of the flower; **F**. Apical view of the flower; **G**. Fruiting branch [photos: **A–G** by X. L. Hou].

##### Description.

Shrubs or small trees, up to 8 m tall. Annual branches reddish brown, pubescent, 1–1.5 mm in diam.; older branches retaining some indumentum, longitudinally striate, without lenticels. Petiole 1–1.5 × 1 mm, indumentum as on branchlets; leaf blades chartaceous, ovate-elliptic or ovate-oblong, 5–11 × 2.2–4 cm, apex acuminate to obtuse, base subrounded, asymmetric; adaxial surface glabrous, midvein and lateral veins nearly flat; abaxial surface glabrous, lateral and reticulate veins inconspicuous; lateral veins (10–)12–14 per side, arched-ascending at ca. 60° from midvein, anastomosing 2–3 mm from margin. Inflorescences axillary, 1–2(–3)-flowered; flowers bisexual, pale purplish red or yellowish green; peduncle 1–3 mm long; bracts 1–2, ovate, ca. 1 × 0.5 mm, pubescent; pedicel 10–15 × 0.4 mm, glabrous, with 1 bracteole at the middle to lower part, bracteole ovate, ca. 1 × 0.5 mm, pubescent. Sepals 3, connate at base, broadly ovate-triangular, 0.5 × 1 mm, abaxially puberulent, adaxially glabrous, persistent. Petals 6, in 2 whorls of 3; outer petals broadly ovate, 1.5–2 × 1.5–2 mm, abaxially pubescent, margin more densely so, adaxially glabrous; inner petals broadly ovate, ca. 4 × 4 mm, leathery when dry, ca. 0.4 mm thick, apex acute, abaxially very sparsely pubescent, margin more densely so, adaxially glabrous, with a transverse crescent-shaped gland at base. Stamens 12–30, 3–4-seriate, 0.8 × 0.8 mm; connective apex obtusely pointed, slightly exceeding thecae; thecae ca. 0.5 mm long. Carpels 5–19, ca. 1.5 mm long; ovary glabrous, ca. 1 mm long; stigma narrowly ellipsoid, glabrous, ca. 0.5 mm long. Torus conical, greyish yellow, pubescent. Fruiting peduncle 1–3 × 1 mm; fruiting pedicel 10–15 × 0.8 mm. Monocarps (1–)3–15, ellipsoid, 8–10 × 6–8 mm, glabrous, with inconspicuous warts; stipes 5–6 × 1 mm, with inconspicuous tubercles. Seed 1, 7–9 × 5–7 mm.

##### Phenology.

Flowering from February to October; fruiting from May to December.

##### Distribution.

Southern China (southwestern Guangxi and southeastern Yunnan).

##### Habitat.

Sparse or secondary forests on limestone mountains at elevations of 100–500 m.

##### Notes.

Li (1943) reported *Orophea anceps* as a new record of *Orophea* for Guangxi Province, based on a single specimen, *S. P. Ko 55657*, which in fact belongs to *Miliusa glochidioides*. This misidentification led Tsiang and Li (1979) to place *Miliusa glochidioides* in *Orophea*. Li and Gilbert (2011) subsequently reinstated *Miliusa glochidioides* as a distinct species within *Miliusa*.

##### Selected specimens examined.

**Guangxi • Bama County**, 13 Jun 2008, Z. Zhong 1468 (PE); • ibid, 13 Jun 2008, Z. Zhong 1479 (PE); • **Daxin County**, 1 Oct 2009, X. L. Hou 9100108 (AU); • ibid, 1 Oct 2009, X. L. Hou 9100109 (AU); • ibid, 2 Oct 2009, X. L. Hou 9100203 (AU); • ibid, alt. 335 m, 22.5933°N, 106.7675°E, 1 Apr 2017, X. L. Hou & R. P. Zhang 90458 (AU); • **Jingxi County**, 20 Aug 2008, Z. Zhong 2939 (PE); • ibid, 24 Oct 2008, Z. Zhong 4553 (PE); • **Longjin County**, 8 Dec 1957, P. X. Tan 57307 (FJSI); • ibid, 19 Oct 1958, H. Q. Li 40167 (FJSI); • **Longzhou County**, 11 Aug 2008, Z. Zhong 2560 (PE); • ibid, 11 Sept 1986, J. Bei 1439 (PE); • ibid, 15 Oct 2008, Z. Zhong 3935 (PE); • ibid, 22 May 2008, Z. Zhong 977 (PE); • ibid, 27 Sept 2009, X. L. Hou 9092706 (AU); • ibid, 27 Sept 2009, X. L. Hou 9092710 (AU); • ibid, 28 Sept 2009, X. L. Hou 9092802 (AU); • ibid, 28 Sept 2009, X. L. Hou 9092803 (AU); • **Wuming County**, 10 May 2008, Z. Zhong 56 (PE); • **Yangshuo County**, alt. 1350 m, 28 Nov 1963, Z. Z. Chen 53414 (KUN). **Yunnan • Funing County**, 11 Oct 1965, S. Z. Wen 79 (KUN); • **Malipo County**, alt. 152 m, 22.9796523°N, 104.7929888°E, 31 Jul 2025, Y. J. Li 382(AU); **Cambodia**: **Samrongtong**, alt. 400–600 m, May 1870, L. Pierre 737B (P, K).

#### 2. Miliusa horsfieldii

Taxon classificationPlantaeMagnolialesAnnonaceae

(Benn.) Pierre, Fl. Forest. Cochinch. 3: t. 38 (1881)

C2FA0FBD-6C36-5EA5-A4B4-90441374CDA5

Fig. 3

Miliusa horsfieldii (Benn.) Pierre, Fl. Forest. Cochinch. 3: t. 38 (1881); P. T. Li & M. G. Gilbert in Fl. China 19: 680 (2011); Fl. Thailand 16(1): 177 (2022). – Saccopetalum horsfieldii Benn., Pl. Jav. Rar. (2): 165, t. 35 (1840). Type: Indonesia. Java, Prov. Banyumas, 1814, T. Horsfield s.n. (lectotype: BM! [BM000554019], designated by Jessup (1988); isolectotype: BM! [BM000554017]).
Miliusa prolifica (Chun & F. C. How) P. T. Li, Guihaia 13 (4): 315 (1993); P. T. Li & Z. K. Li in High. Pl. China 3: 167, t. 257 (2000). – Alphonsea prolifica Chun & F. C. How, Acta Phytotax. Sin. 7: 1, t. 1 (1958). – Saccopetalum prolificum (Chun & F. C. How) Y. Tsiang in Acta Phytotax. Sin. 9: 380, pl. 37 (2) (1964); Y. Tsiang et P. T. Li in Fl. Hainan 1: 254 (1964); P. T. Li in Icon. Cormophyt. Sin. 1: 811, t. 1622 (1972); Y. Tsiang & P. T. Li in Fl. Reipubl. Popularis Sin. 30 (2): 43, t. 17 (1979). Type: China. Hainan: Yai Hsien, Lofung, 5 Jul 1933, *F. C. How 70951* (holotype: IBSC! [IBSC003387]; isotypes: A! [A00056747], B! [B100591455], K! [K001089921], NY! [NY00334809], P! [P00160917], US! [US00478958]).
Alphonsea mollis sensu Merr. & Chun in Sunyatsenia 2: 230. (1935), non Dunn.

##### Chinese name.

囊瓣野独活 Náng Bàn Yě Dú Huó.

**Figure 3.**
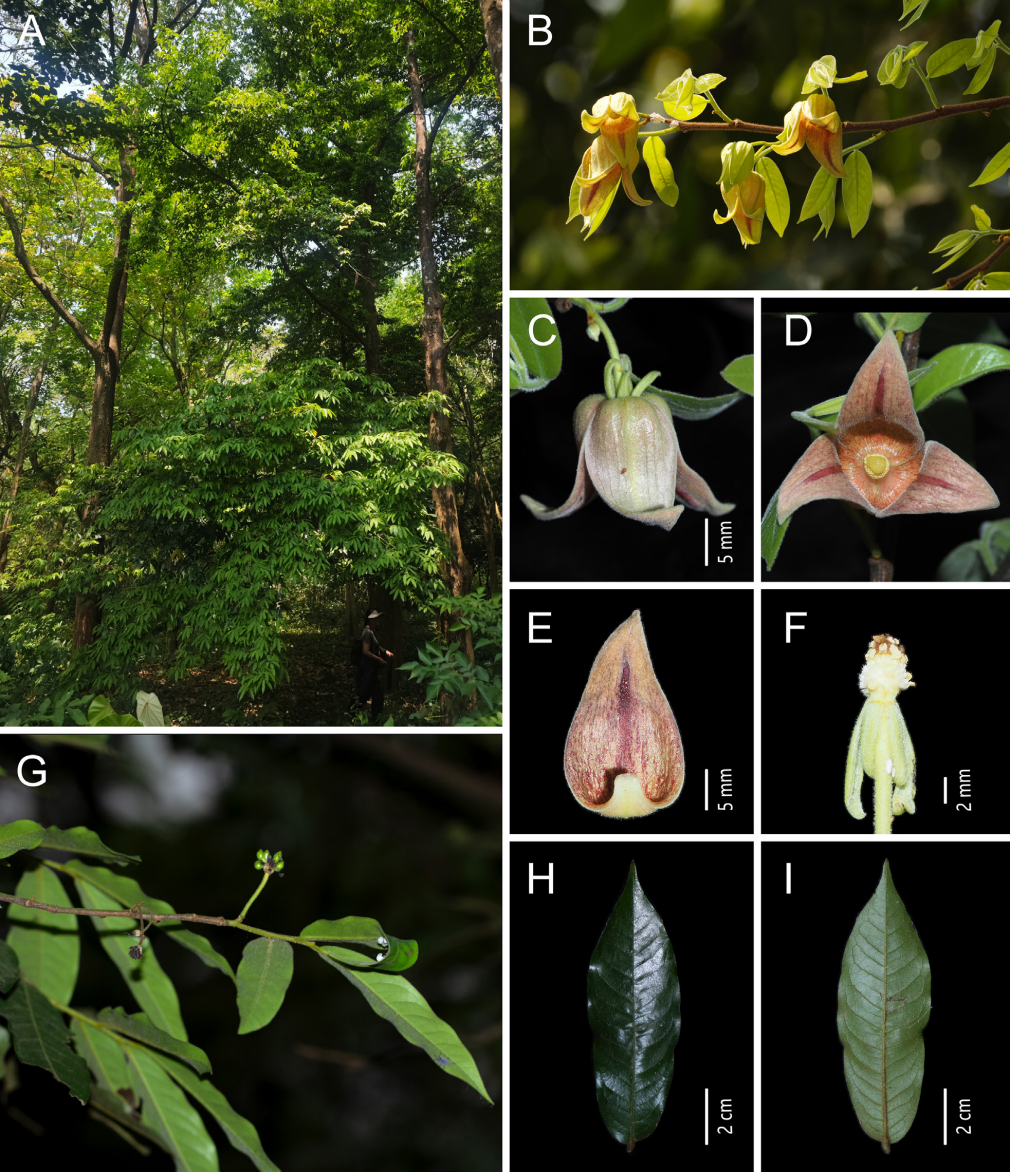
*Miliusa horsfieldii* (Benn.) Pierre. **A**. Plant; **B**. Flowering branch; **C**. Lateral view of the flowers; **D**. Bottom view of the flower; **E**. Abaxial view of the inner petal; **F**. Flower without inner petals; **G**. Fruiting branch; **H**. Adaxial view of the leaf blade; **I**. Abaxial view of leaf the blade [photos: **A** by Z. Y. Shi **B** by W. F. Hu **C–F** by Y. S. Chen **G–I** by X. L. Hou].

##### Description.

Tree up to 25 m tall, to 50 cm in diam.; bark pale yellow; phloem yellowish brown, slightly pungent. Deciduous in winter, though a few leaves often persist; new leaves emerging at anthesis. Annual branches 1.5–2 mm in diam., densely covered with spreading yellowish brown hairs ca. 1 mm long; older branches sparsely pubescent to glabrous, sparsely brown-lenticellate. Petiole 2–4 × 1.5 mm, densely spreading yellowish brown pubescent; leaf blades chartaceous, ovate-oblong to oblong, occasionally elliptic or obovate-oblong, 4–13 × 2–4.5 cm, base rounded, slightly asymmetric, apex acuminate or acute; adaxial surface sparsely pubescent, more densely so on the midvein; abaxial surface densely villous; midvein and lateral veins flat adaxially, raised abaxially; lateral veins 10–14 per side, slender, slightly conspicuous adaxially and abaxially, arched-ascending at 40°–45° from midvein, anastomosing 4–5 mm from margin; tertiary veins inconspicuous. Inflorescences on older or current-year branches, axillary, 1- or 2-flowered; flowers bisexual, initially yellowish green, becoming light to dark red at maturity, densely villous; peduncle 2–3 × 1–1.5 mm; pedicel 15–30 × 1 mm, reflexed, sparsely villous. Sepals 3, free, narrowly ovate, 5–6(–10) × 1.5–2 mm, densely pubescent on both surfaces. Petals 6, in 2 whorls of 3, both surfaces densely pubescent; outer petals narrowly lanceolate, 7–10(–13) × 1.5–2 mm, reflexed; inner petals ovate, 20–30 × 10–15 mm, base saccate, inner surface often red, margins more or less appressed, apex spreading and slightly recurved, usually with narrow red stripes along the midvein on the inner surface. Stamens 50–60 in 5–6 whorls, ca. 1 mm long; connective incurved, apically pointed. Carpels 25–35, densely villous; ovary ca. 1 mm long; stigma narrowly ellipsoid, ca. 0.5 mm long, glabrous. Torus ellipsoid, densely white pubescent. Fruiting peduncle 3 × 2 mm; fruiting pedicel 18–25 × 2 mm; stipe 10–18 × 1 mm, pubescent. Monocarps 15–20, subglobose, 1–2 cm in diam., puberulent, initially yellowish green, turning dark red at maturity; seeds 2–8 per monocarp, reniform, with one or two sides flattened, ca. 11 × 5 mm.

##### Phenology.

Flowering from March to April; fruiting from July to August.

##### Distribution.

China (Hainan: Baoting, Changjiang, Dongxian, Lingshui, and Lehui; Guangdong: Yangchun; Guangxi: Longzhou) and South and Southeast Asia, extending to Australia (India, Myanmar, Laos, Thailand, Malaysia, Indonesia, the Philippines, and Australia); also cultivated in Guangzhou.

##### Habitat.

Dense valley forests at elevations of 300–1000 m.

##### Notes.

This species was transferred to *Saccopetalum* on the basis of its combination of non-connate inner petals and up to eight ovules arranged in two rows. Sinclair (1955) argued that characters such as ovule number and the sac-like form of the inner petals are shared by *Saccopetalum* and *Miliusa*, thereby supporting Baillon’s (1867) view that *Saccopetalum* should be reduced to *Miliusa*. This interpretation was subsequently followed by Fries (1959), Ban (2000), Mols and Kessler (2003b), and Li and Gilbert (2011). When Tsiang and Li (1964) recombined *Alphonsea prolifica* Chun & F. C. How as *Saccopetalum prolificum* (Chun & F. C. How) Y. Tsiang, they designated F. C. How 71794 as the type; however, this typification was superfluous. Guangxi represents a newly recorded distribution for this species in China.

##### Selected specimens examined.

**Guangdong • Yangchun County**, alt. 200 m, 24 Nov 1991, H. G. Ye & N. Liu 3133 (IBSC); • ibid, 2 Nov 2002, H. G. Ye 7648 (IBSC). **Guangxi • Longzhou County**, Sept 1973, Y. T. Zhang Q11(3) (PE). **Hainan • Baoting County**, alt. 330 m, 10 Apr 1935, F. C. How 71794 (IBK, IBSC); • ibid, alt. 150 m, 13 Jun 1958, Q. J. Chen 89544 (IBSC); • ibid, 25 Sept 2012, X. L. Hou & T. X. Sun et al. 12092510 (AU); • ibid, 18.7081°N, 109.6417°E, 9 Feb 2011, X. L. Hou & T. X. Sun 11020904 (AU); • **Changjiang County**, alt. 600 m, 22 Jun 1982, G. A. Fu 2842 (IBSC); • **Dongfang City**, 21 Jun 1957, Y. T. Lin 90389 (IBSC); • **Ledong County**, 2 Oct 2012, X. L. Hou & T. X. Sun et al. 12100203 (AU); • ibid, 7 Apr 2013, X. L. Hou 2013040708 (AU); • ibid, 11 Jun 2016, X. L. Hou & W. W. Xin et al. 71 (AU); • ibid, 24 Apr 2025, Y. J. Li 254 (AU); • **Lingshui County**, alt. 400 m, 31 Oct 1981, G. A. Fu 152967 (IBSC); • ibid, 10 Dec 1986, F. W. Xing & Z. X. Li 44 (IBSC); • ibid, 10 Dec 1986, F. W. Xing & Z. X. Li s.n. (IBSC); • **Qionghai City** (Lehui County), 13 May 1935, F. C. How 72328 (IBK, IBSC); • **Wanning City**, 10 Apr 1935, F. C. How 71794 (IBK); • **Yazhou District, Sanya City**, 5 Jul 1933, F. C. How 70951 (IBSC); • ibid, 18.392375°N, 109.646275°E, 35 Jan 2011, X. L. Hou & T. X. Sun 11012808 (AU).

#### 3. Miliusa aurilaveoides

Taxon classificationPlantaeMagnolialesAnnonaceae

X.L.Hou & Z.Y.Shi sp. nov.

4D6F0CA3-3B37-5580-8B9C-DFB7438D2B68

urn:lsid:ipni.org:names:77378908-1

Fig. 4

##### Chinese name.

洗耳球野独活 Xǐ Ěr Qiú Yě Dú Huó.

**Figure 4.**
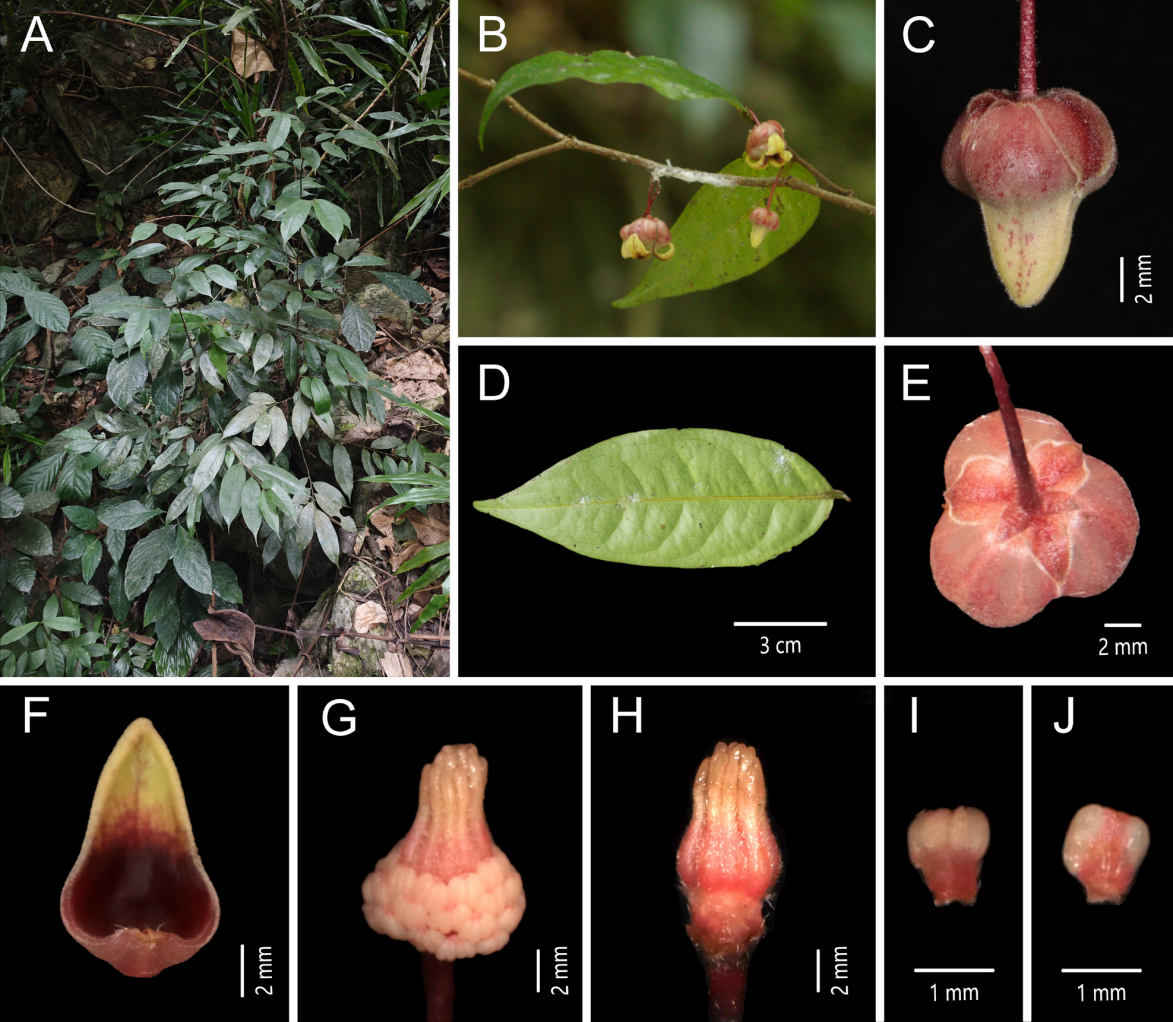
*Miliusa aurilaveoides* X. L. Hou & Z. Y. Shi. **A**. Plant; **B**. Flowering branch; **C**. Lateral view of the flower; **D**. Abaxial view of the leaf blade; **E**. Apical view of the flower; **F**. Adaxial view of the inner petal; **G**. Flower without petals and sepals; **H**. Gynoecium; **I**. Abaxial view of the stamen; **J**. Adaxial view of the stamen [photos: **A** by Z.Y. Shi **B–J** by X. L. Hou].

##### Type material.

China • Yunnan Province: Tianbao Town, Malipo County, alt. 123 m, 22.9828, 104.7839, 12 Apr 2024, *Y. J. Li 268* (holotype: AU; isotype: AU).

##### Diagnosis.

This species resembles *Miliusa balansae* but can be readily distinguished by its flower buds, which are abruptly constricted at the middle and resemble a bulb syringe (vs. conical), by its pedicels 1.5–2.2 cm long (vs. 4–7 cm), and by its outer petals 4–5 mm long (vs. 2–2.5 mm).

##### Description.

Shrubs or small trees, 2–3 m tall. Annual branches ca. 1.5 mm in diam., densely covered with spreading greyish yellow hairs 0.3–0.5 mm long, without lenticels; older branches sparsely pubescent to nearly glabrous. Petiole 3–4 mm long, adaxially grooved, abaxially transversely wrinkled, slightly swollen, turning from green to brown, densely greyish yellow pubescent; leaf blades chartaceous, ovate-oblong to narrowly ovate-oblong, 9–17 × 3–5.5 cm; apex acuminate, acumen 1.5–2.5 cm long; base obtuse to rounded, slightly asymmetrical; midvein adaxially impressed, abaxially raised; lateral veins adaxially inconspicuous, abaxially slightly raised, 9–12 per side, arched-ascending at 55°–65° from midvein, anastomosing 3–4 mm from margin; intersecondary veins well developed, 5–8 per side; tertiary veins forming a conspicuous reticulation; adaxial surface dark green, glabrous except for the pubescent midvein; abaxial surface pale green, with appressed white hairs 0.3–0.5 mm long and dense transparent glandular dots. Inflorescences axillary on current-year or leafless older branches, usually 1(–2)-flowered; flowers bisexual. Flower bud narrowly conical in the upper half, abruptly constricted at the middle, lower half saccate, resembling a bulb syringe in vivo, apex yellow, base pale purplish red, ca. 1.5 cm in diam. at anthesis; peduncle ca. 0.5–1 mm long, with 1 bract, broadly ovate, 1 × 1 mm; pedicel 1.5–2.2 cm long, ca. 0.5 mm in diam. at the middle, gradually thickening distally, purplish red in vivo, sparsely covered with spreading white hairs ca. 0.3 mm long, with a single basal bracteole, ovate, 1 × 0.5 mm, sparsely puberulent. Sepals 3, free, pale red in vivo, ovate-triangular, 3 × 1.6 mm, abaxially densely pubescent with hairs ca. 0.3 mm long, ciliate, adaxially glabrous. Petals 6 in two whorls of 3; outer petals free, pale red in vivo, variable in shape and size, ovate to broadly obovate, 4–5 × 2.5–5 mm, apex shortly acuminate, abaxially white pubescent and ciliate with hairs ca. 0.3 mm long, adaxially glabrous; inner petals ovate, 17–19 × 10 mm, margins appressed from base to ca. ± 2/3 of the length, upper 1/3 strongly reflexed, pale yellow, apex acute; abaxial surface with scattered purple streaks; adaxial surface with a dark red patch extending from the midvein; base saccate, red, with 7 distinct veins and conspicuous translucent “windows” between veins; median region with a dark red patch accompanied by tuberculate protuberances along the midvein; upper 1/3 of both surfaces puberulent. Stamens in ca. 5 whorls, 42–50, 1.2 × 1 mm; connective pale red in vivo, apex obtuse; anthers 2-locular, white, oblong, ca. 0.7 mm long; filaments conspicuous, ca. 0.4 mm long. Carpels 15–25, ca. 3 mm long; ovary oblong, ca. 1.5 mm long, pale red *in vivo*, white pubescent; stigma cylindrical, ca. 1.5 mm long, glabrous; ovules 2 per carpel. Torus conical, densely white pubescent, hairs ca. 0.5 mm long. Fruit unknown.

##### Phenology.

Flowering recorded in April; fruiting not observed.

##### Distribution.

Known only in Yunnan, China (Malipo County).

##### Habitat.

Understory of dense, moist forests on limestone mountains.

##### Selected specimens examined.

**Yunnan Province • Malipo County**, Tianbao Town, alt. 123 m, 22.98278°N, 104.78389°E, 8 Jan 2025, *Z. Y. Shi 2025010806* (AU).

##### Etymology.

The specific epithet “aurilaveoides” refers to the morphology of the flower buds, which have a narrowly conical upper portion, an abruptly constricted middle, and an urn-shaped base, collectively resembling an aurilave (ear syringe). The suffix “-*oides*” denotes resemblance.

#### 4. Miliusa longicarpa

Taxon classificationPlantaeMagnolialesAnnonaceae

Z. Y. Shi & X. L. Hou, Phytotaxa 666(3): 209–215 (2024)

B9BED949-7DC9-56CF-BE2B-196CAB3C8F54

Fig. 5

Miliusa longicarpa Z. Y. Shi & X. L. Hou, Phytotaxa 666(3): 209–215 (2024). Type: China. Yunnan: Cangyuan County, Banhong Xiang, 2 August 2021, Z. Y. Shi 162 (holotype: AU!; isotypes: AU!).

##### Chinese name.

长果野独活 Cháng Guǒ Yě Dú Huó.

**Figure 5.**
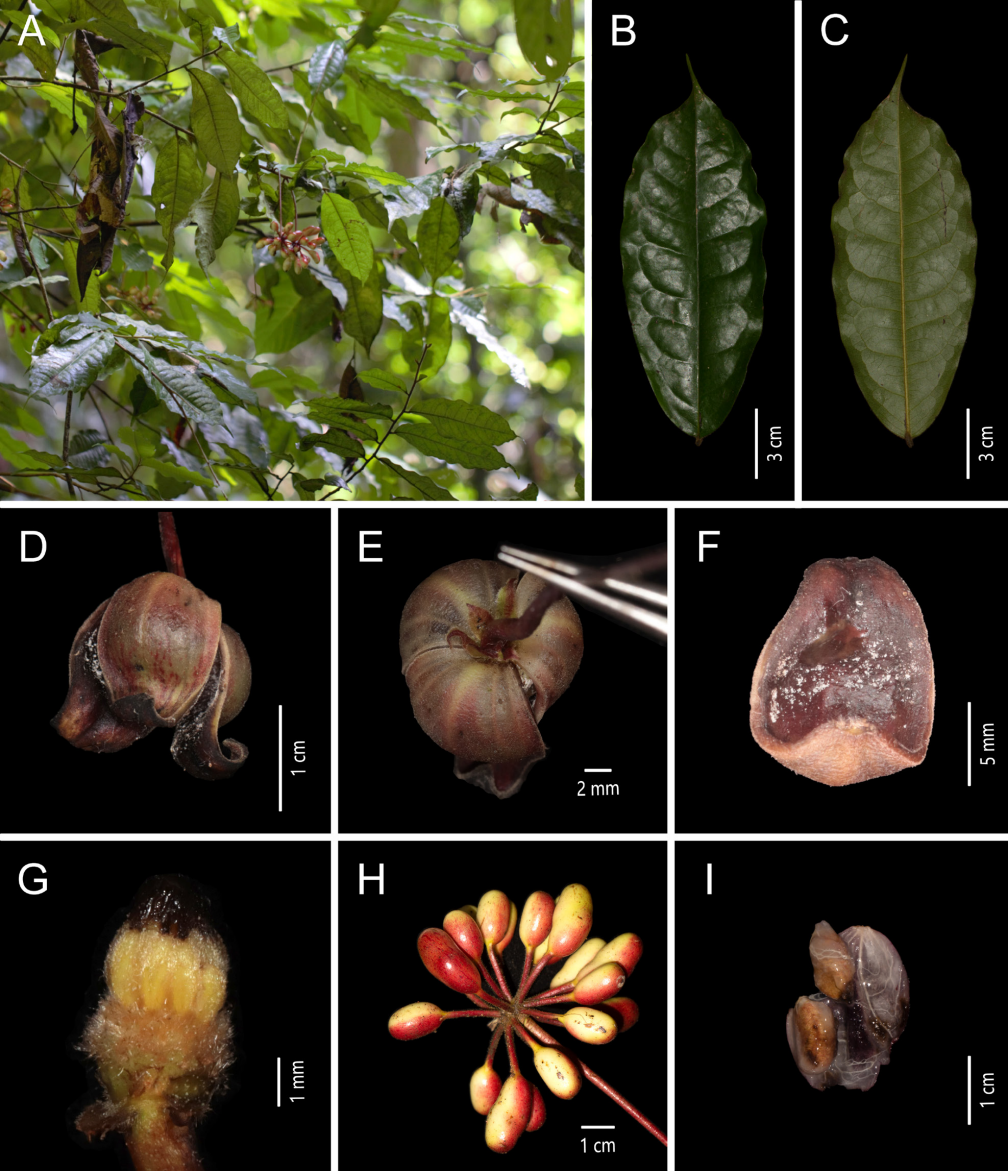
*Miliusa longicarpa* Z. Y. Shi & X. L. Hou. **A**. Fruiting branch; **B**. Adaxial view of the leaf blade; **C**. Abaxial view of the leaf blade; **D**. Flowers; **E**. Apical view of the flower; **F**. Adaxialview of the inner petal; **G**. Gynoecium; **H**. Fruit; **I**. Seeds [photos: **A–C, H, I** by Z.Y. Shi **D–G** by Z.Y. Zhang].

##### Description.

Small trees up to 7 m tall; branches terete. Annual branches pubescent, 1.5 mm in diam. Petiole 3–4 mm long, pubescent, slightly grooved adaxially; leaf blades elliptic, 9.5–21.0 × 4.2–6.1 cm, glabrous above, pubescent below, base rounded, symmetric, apex acuminate to caudate, acumen usually 15–21 mm long; margin entire; midvein sunken above, almost glabrous, raised below, pubescent with straight, hairs 0.4–0.6 mm long; lateral veins 10–13 on each side of midvein, straight, ascending, forming loops near margin, angle with midvein 51°–60° (at middle part of leaf blade), prominent below; intersecondary veins well developed. Inflorescences axillary, 1-flowered; flowers bisexual; peduncles 1.0–2.0 mm long, pubescent; bracts 3, broadly triangular, 0.5–1.2 × 0.6–1.0 mm, pubescent; pedicels 5.6–8.3 cm long, 0.5–0.8 mm in diam., sparsely pilose; bracts 1–3, ovate, ca. 0.5 mm long. Sepals 3, free, green tinged with pale red, ovate-triangular, 3 × 2 mm, abaxially sparsely pubescent, adaxially glabrous, persistent. Petals 6, in 2 whorls of 3; outer petals green tinged with pale red, triangular, 3.5–4.1 × 1.9 mm, sparsely pubescent abaxially, glabrous adaxially; inner petals 3, dark red, ovate, 16–18 × 8–10 mm, sparsely pubescent abaxially, glabrous adaxially, margins appressed from base to middle, upper 1/3 strongly reflexed, pale yellow, apex acute; abaxial surface with scattered purple streaks; adaxial surface with a dark red patch at the middle; base saccate, with 5–7 distinct veins and inconspicuous translucent “windows” between veins. Stamens in 6–7 whorls, ca. 40–50, 1.2 × 1 mm; anthers 2-locular, oblong, ca. 0.7 mm long; filaments conspicuous, ca. 0.5 mm long. Carpels 22–28, 2.5 mm long; ovary oblong, ca. 1.5 mm long, densely pubescent; stigma linear-oblong, ca. 1.0 mm long, glabrous, with viscous exudate; ovules 2 per carpel. Torus globular, densely pubescent; fruiting pedicels 7.8–12.0 cm long, 1.4–1.6 mm in diam., gradually thickening upwards, pendulous, almost glabrous. Monocarps 10–20, ellipsoid, 10–19 × 6–10 mm, smooth, almost glabrous, constricted at the middle when 2-seeded, apex not apiculate, red at maturity; stipes 1.1–1.6 cm long, slender, red, pubescent with straight hairs 0.3–0.5 mm long. Seeds 1 or 2 per monocarp, ellipsoid, ca. 10 × 6 mm.

##### Phenology.

Flowering in April; fruiting in August.

##### Distribution.

Known from Yunnan, China (Cangyuan, Jinping).

##### Habitat.

Understory of dense, humid, shaded forests at elevations of 600–800 m.

##### Selected specimens examined.

**Yunnan • Cangyuan County**, Banhong Xiang, valley forest, alt. 661 m, 23.26917°N, 99.07194°E, 2 Aug 2021, Z. Y. Shi 161 (AU); • ibid, 22 Apr 2024, G. M. Yang 01 (AU); • ibid, 22 Apr 2024, F. Y. Zhong 212 (AU); • ibid, 24 May 1974, Y. H. Li 11603 (HITBC); • ibid, 28 Jun 1974, Y. H. Li 12555 (HITBC); • ibid, dense forest with wet, shaded slopes along streams, 780–800 m, 2 Jun 1974, Y. H. Li 11867 (HITBC, IBSC, KUN); • **Jinping County**, alt. 1500 m, 21 Aug 1951, P. Y. Mao 441 (HITBC, IBSC, KUN, PE, WUK).

#### 5. Miliusa balansae

Taxon classificationPlantaeMagnolialesAnnonaceae

Finet & Gagnep., Bull. Soc. Bot. France 53 (Mém. 4): 149 (1906)

A8A8E855-0A76-5917-9B02-6F80836176EA

Fig. 6, Table 1

Miliusa balansae Finet & Gagnep., Bull. Soc. Bot. France 53 (Mém. 4): 149 (1906); P. T. Li in Fl. Guangdong 2: 16, t. 11(6–9) (1987); S. H. Yuan in Fl. Yunnan 5: 16 (1991); P. T. Li & Z. K. Li in High. Pl. China 3: 166, t. 256 (2000); N. T. Ban, Fl. Viet. 1: 308 (2000); P. T. Li & M. G. Gilbert in Fl. China 19: 680 (2011). Type: Vietnam. Tonkin: Son Tay, forêt du Mont Bavi, March 1887, B. Balansa 3140 (holotype: P! [P00160836]; isotype: P! [P00432376]).
Miliusa chunii W. T. Wang in C. Y. Wu et W. T. Wang, Acta Phytotax. Sin. 6: 202 (1957); Y. Tsiang et P. T. Li in Fl. Hainan 1 (1964); P. T. Li in Icon. Cormophyt. Sin. 1: 812, t. 1624 (1972); Y. Tsiang et P. T. Li in Fl. Reip. Popul. Sin. 30 (2): 42, t. 16 (9–12) (1979); P. T. Li in Fl. Guangxi 1: 122, t. 52 (1991). – Miliusa filipes Merr. & Chun, Sunyatsenia 2: 223–224, fig. 22 (1935), nom. illegit., non Ridl. in Journ. Fed. Mal. States Mus. 10: 81(1920); Y. Tsiang in Bull. Bot. Soc. China 2 (3): 684, t. 3 (1935); Chen, R., Classification of Chinese Trees 327 (1937); Y. Tsiang et P. T. Li in Fl. Reip. Popul. Sin. 30 (2): 42, t. 16 (9–12) (1979). Type: China. Hainan: Yaichow, 15 March 1933, *F. C. How 70357* (holotype: NY! [NY00026114]; isotypes: A! [A00039451], B! [B100591460], IBK! [IBK00190118T, IBK00190119T], IBSC! [IBSC0079066], K! [K001089934], NAS! [NAS00321907], P! [P00411062], PE! [PE01187094], SYS! [SYS00095588T], US! [US01669487]).

##### Chinese name.

野独活 Yě Dú Huó.

**Figure 6.**
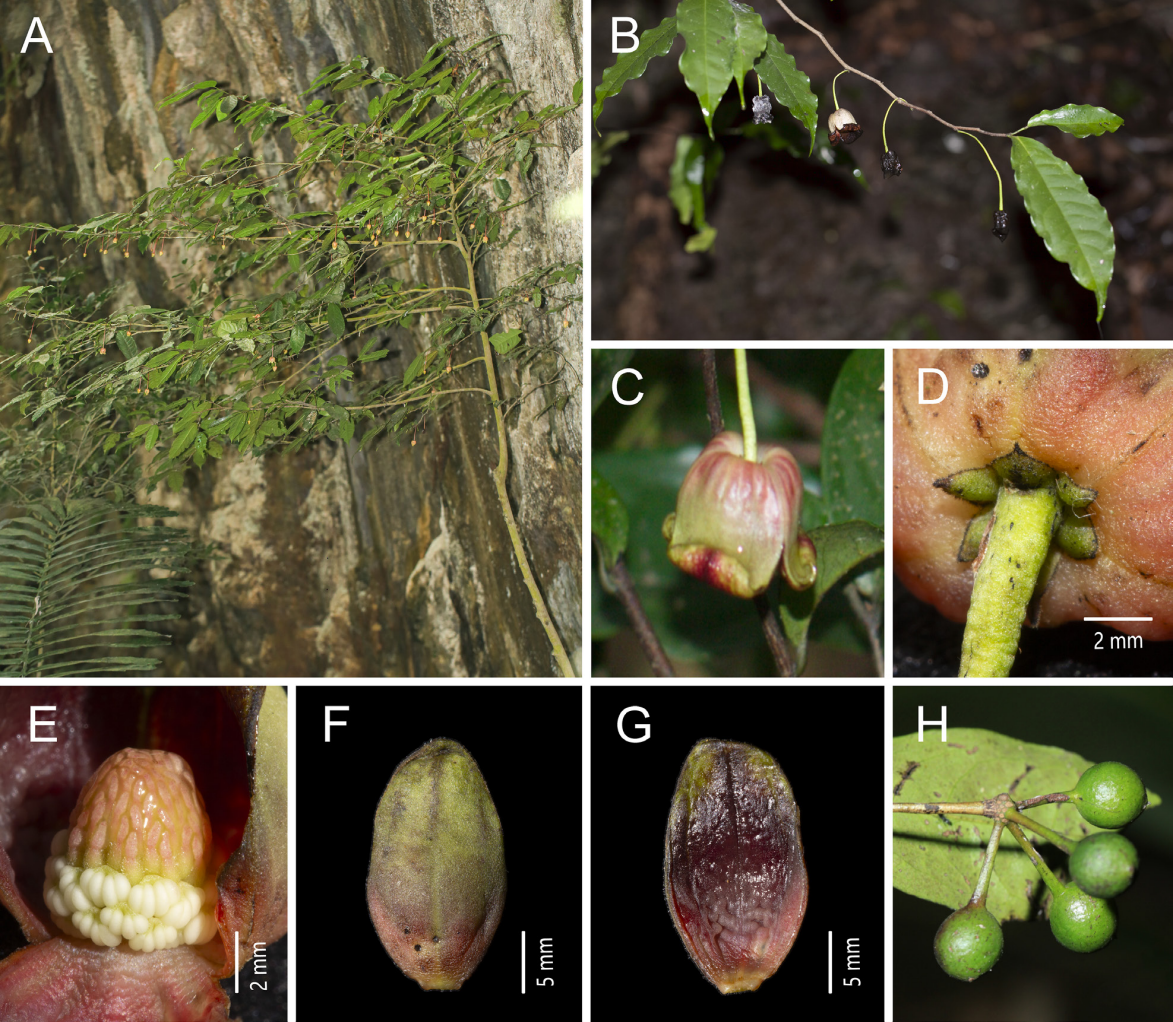
*Miliusa balansae* Finet & Gagnep. **A**. Plant; **B**. Flowering branch; **C**. Lateral view of flowers; **D**. Apical view of the flower; **E**. Androecium and gynoecium; **F**. Abaxial view of the inner petal; **G**. Adaxial view of the inner petal; **H**. Fruit [photos: **A–H** by X. L. Hou].

**Table 1.** The comparison of the diagnostic characters of *Miliusa sinensis* and *M. balansae*.

Characters	*Miliusa sinensis*	*M. balansae*
Sepals	ovate-triangular, 2.5–3 mm long, densely pubescent	broadly ovate-triangular, 2 mm long, glabrous or sparsely minutely pubescent
Outer petals	narrowly ovate-triangular, 3–4 mm long, densely pubescent	ovate-triangular, 2–2.5 mm long, glabrous or sparsely minutely pubescent
Inner petals	ovate, 10–18 × 7–10 mm, densely minutely pubescent	ovate, 15–25 × 10–15 mm, glabrous or sparsely minutely pubescent
Pedicel	0.4–0.6 mm in diam., spreading yellow pubescence, hairs ca. 0.5 mm long	0.5–1 mm in diam., glabrous or sparsely minutely pubescent, hairs 0.1–0.2 mm long
Monocarps	9–14 × 8–10 mm	12–22 × 10–14 mm
Branches indumentum	densely spreading yellowish-gray pubescence, hairs ca. 0.5 mm long	glabrous or minutely puberulent, hairs 0.1–0.2 mm long

##### Description.

Small trees, 2–5 m tall. Branches 1–1.5 mm in diam., densely to sparsely pubescent, becoming glabrescent, without lenticels. Petiole 2–4(–5) × 1.5 mm, sparsely pubescent; leaf blades chartaceous, variable in shape and size, ovate-elliptic, ovate-oblong, or obovate-elliptic, 8–19 × 3–6 cm, apex acuminate with a tip 5–20 mm long, base rounded to broadly cuneate; adaxial surface glabrous, midvein slightly impressed; abaxial surface initially with appressed short hairs, gradually glabrescent, midvein and lateral veins raised, tertiary veins clearly visible; lateral veins 10–12 on each side, arched-ascending at 60°–80° from the midvein, anastomosing ca. 6–9 mm from the margin. Inflorescences axillary, 1(–2)-flowered; flowers bisexual; peduncle ca. 1 mm long; pedicel 40–65(–90) × 0.8–1 mm, glabrous, with 1 bracteole at the middle and 1 at the base, bracteoles ovate, 1 × 0.5 mm. Sepals 3, free, broadly ovate-triangular, 2 × 1.5–2 mm, margin and abaxial surface sparsely puberulent with hairs 0.1–0.2 mm long. Petals 6, in 2 whorls of 3; outer petals ovate-triangular, 2–2.5 × 1.5 mm, glabrous or puberulent, margin ciliate with hairs 0.1–0.2 mm long; inner petals broadly ovate, 15–25 × 10–15 mm, glabrous, yellowish green in bud, gradually turning purplish red or light red at anthesis, margins appressed from base to about the midpoint, upper ca. 1/4–1/3 strongly reflexed, lower half saccate, with 5–7 veins and conspicuous red tuberculate protuberances along the veins. Stamens in 7 or 9 whorls, 50–75, obovate, 1 × 1 mm; connectives apically mucronate. Carpels 50–60, ca. 1.5 mm long; ovary ovoid, ca. 1 mm long, sparsely pubescent; stigma narrowly ovoid, ca. 0.5 mm long, puberulent; ovules 2(–3) per ovary. Torus cylindrical. Fruiting peduncle ca. 2 × 2 mm; fruiting pedicel 50–75(–100) × 1.5–2.5 mm, glabrous, longitudinally striate. Monocarps 12–35, red to black at maturity, berry-like, asymmetrically attached at base, globose or ellipsoid-globose, 12–22 × 10–14 mm, apex apiculate, tuberculate or smooth, constricted between seeds; stipes 15–30 × 1 mm, glabrous. Seeds 1–2(–3) per monocarp, ellipsoid-globose, slightly compressed, 8 × 6 mm.

##### Phenology.

Flowering from March to July; fruiting July to the following March.

##### Distribution.

Widely distributed in southern China (Guangdong: Xinyi, Yangchun; Guangxi: Fangcheng, Longjin, Zhenbian, Yongfu, Jingxi, Du’an, Yangshuo, Longzhou, Napo, Mingjiang, Lipu, Nandan; Guizhou: Libo; Hainan: Wanning, Sanya, Baoting, Qionghai, Qiongzhong, Lingshui; Yunnan: Mengla, Simao, Funing) and extending southward into Vietnam.

##### Habitat.

Montane forests and shrublands in valleys at elevations of 100–2100 m.

##### Notes.

**Figure 7.**
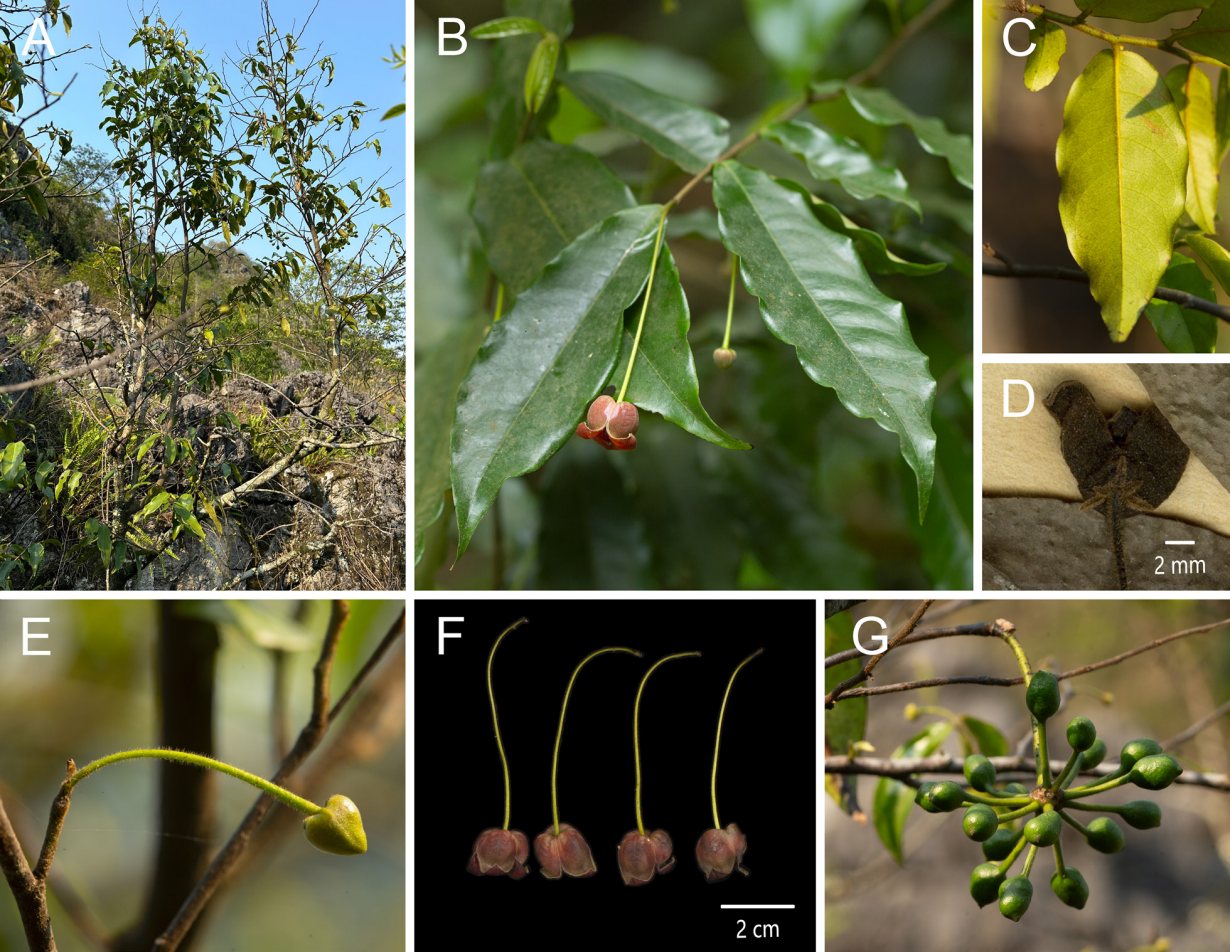
*Miliusa sinensis* Finet & Gagnep. **A**. Plant; **B**. Flowering branch; **C**. Twig (showing dense hairs on branch and leaves); **D**. Flowers (showing sepals and outer petals, isotypes P [P00432372]); **E**. Flower buds; **F**. Flowers; **G**. Fruit [photos: **A–C, E–G** by X. L. Hou].

This species is widely distributed in China and occurs in large populations and is therefore considered common. It is particularly frequent in Guangxi and Hainan, with its range extending north to Duyun City (25.85°N), placing it among the few Annonaceae species in China that reach such a northerly latitude. It primarily inhabits limestone mountains, preferring humid climates but tolerating relatively low temperatures. It occurs in montane forests up to alt. 2100 m and is consequently one of the few Annonaceae species in China recorded above 2000 m. Its broad distribution encompasses markedly heterogeneous habitats, which is reflected in considerable morphological variation in leaves, flowers, and fruits. Key diagnostic characters include: plants glabrous or finely pubescent throughout; sepals broadly ovate-triangular, 2 × 1.5–2 mm; outer petals ovate–triangular, 2–2.5 × 1.5 mm; inner petals ovate, relatively large, 15–25 × 10–15 mm; pedicels elongate, 40–65(–90) × 0.8–1 mm (Fig. 7).

##### Selected specimens examined.

**Guangdong • Xinyi City**, 7 May 1931, X. P. Gao 51429 (IBK, IBSC, KUN, PE, JXU). **Guangxi • Baise County**, alt. 2100 m, 4 Jun 1958, Z. T. Li 600676 (IBK); • **Chongzuo City**, 28 Mar 2015, D. X. Nong & Y. Y. Xie et al. 451402150328005LY (GXMG); • **Dongxing City**, 27 Jun 2019, X. S. Dong & C. D. Pu 450681190627040LY (IBK); • **Du’an County**, 4 Jul 1957, Y. K. Li 01606 (IBK, IBSC, PE); • **Fangcheng County**, 9 Sept 1936, W. T. Tsang 26757 (IBSC); • **Fusui County**, 6 Apr 2008, N. Li & D. Qian et al. 08LN058 (SZG); • **Gongcheng County**, 1 Apr 2015, Gongcheng Team 450332150401013LY (IBK); • **Guanyang County**, 13 Apr 2016, Guanyang Team 450327160413018LY (GXMG); • **Guilin City**, 2 Apr 2020, R. F. Li & D. C. Meng et al. 390 (IBK); • **Hezhou City**, 26 May 2010, N. F. Li & C. H. Lu 22544 (GXMG); • **Huanjiang County**, 3 Dec 2012, Huanjiang Team 451226121203022LY (GXMG, IBK); • **Jingxi County**, 23 Sept 1935, X. P. Gao 55815 (IBK, IBSC); • **Lingchuan County**, 4 May 2015, Z. Y. Zhang & L. Xie et al. 2015158 (PE); • **Lipu County**, L. H. Wei 100150 (IBK); • **Liujiang County**, 4 Apr 2019, Liujiang Team 450221190404025LY (IBK); • **Long’an County**, 6 Mar 2013, Long’an expedition 450123130306010LY (IBK); • **Longjin County**, 5 Oct 1958, H. Q. Li 40088 (IBK, IBSC, FJSI); • **Longzhou County**, 3 Nov 1979, X. H. Lu 8114 (GXMG); • **Luocheng County**, 7 Nov 2013, Luocheng Team 451225131107021LY (GXMG, IBK); • **Luzhai County**, 10 Sept 2019, Luzhai Team 450223190910013LY (GXMG, IBK); • **Nandan County**, 2 Jul 2019, Nandan Team 451221190702021LY (GXMG); • **Napo County**, 4 Sept 2014, D. X. Nong & B. Y. Huang et al. 451026140904041LY (GXMG); • **Ningming County**, 30 May 2013, Y. D. Peng & C. L. Pan et al. 451422130527061LY (GXMG); • Pingle County, 8 Sept 2018, L. Ping 450330180908011LY (IBK); • **Rong’an County**, 6 Aug 2017, Rong’an Team 450224170806031LY (GXMG, IBK); • **Tianlin County**, 29 Nov 1957, Z. D. Nan 5143 (IBK); • **Yangshuo County**, Z. Z. Chen 53260 (IBK); • **Yongfu County**, 4 Aug 2013, Yongfu Team 450326130804032LY (GXMG, IBK); • **Zhongshan County**, 16 Jul 2017, Zhongshan Team 451122170716007LY (GXMG, IBK). **Guizhou • Duyun County**, Oct 1991, Z. Gui 91026 (GZAC); • **Libo County**, 2 Dec 2018, Z. R. Chen GZBGCZR005BS004 (KUN). **Hainan • Baoting County**, 3 Apr 2013, X. L. Hou 2013040308 (AU); • **Ledong County**, 3 Jul 1936, X. Q. Liu 27405 (PE); • **Lingshui County**, 16 Nov 1956, L. Deng 3164 (IBSC, KUN, PE); • **Qionghai County**, alt. 120 m, 28 Jun 1961, Y. Zhong 4494 (IBSC); • **Qiongzhong County**, alt. 60 m, 16 Sept 1957, H. D. Zhang 2291 (IBSC); • **Sanya City**, alt. 640 m, 15 Mar 1933, F. C. How 70355 (IBK, IBSC, NAS, PE, SN); • **Wanning City**, 9 Mar 1995, F. W. Xing 5379 (IBSC); • **Yazhou District**, Sanya City, 25 Sept 1933, Z. Huang 34228 (JXU, IBSC). **Hunan • Jiangyong County**, 17 Apr 1984, Y. T. Xiao 40504 (CSFI). **Yunnan • Funing County**, 16 Oct 1964, Q. A. Wu 9644 (KUN); • **Gejiu City**, 7 Mar 2018, T. Zhang & C. H. Li 18CS16695 (KUN); • **Hekou** County, 23 Apr 2015, X. L. Hou & W. W. Xin 2015042301 (AU); • **Jinghong City**, 6 Dec 1993, H. Wang & B. G. Li 108 (HITBC); • **Maguan County**, 18 Oct 2002, Y. L. Shui & W. H. Chen et al. 31751 (PE); • **Malipo County**, 13 Oct 2010, B. Xiao LuJL342 (KUN); • **Mengla County**, 11 Dec 1982, C. D. Kao 34442 (HITBC); • **Pingbian County**, alt. 3200 m, 15 Mar 1940, X. Wang & X. P. Li 100319 (IBSC); • **Southeast Yunnan**, Dec 1991, H. Wang & H. Zhu 6326 (HITBC); • Southeast Yunnan, Nov 1991, H. Zhu & H. Wang 4824 (HITBC); • **Xichou County**, 5 Oct 1960, S. Z. Wen 188 (KUN). The complete list of specimens is provided in Suppl. material 1.

#### 6. Miliusa sinensis

Taxon classificationPlantaeMagnolialesAnnonaceae

Finet & Gagnep., Bull. Soc. Bot. France 53 (Mém. 4): 151 (1906)

B29877B6-E628-52CD-AAB2-770B1FED1958

Fig. 7, Table 1

Miliusa sinensis Finet & Gagnep., Bull. Soc. Bot. France 53 (Mém. 4): 151 (1906); Levl., Fl. Kouy-Tcheou: 29 (1914); Rehd. in J. Arn. Arb. 10: 191 (1929); C. Y. Wu & W. T. Wang in Acta Phytotax. Sin. 6: 201 (1957); R. E. Fries in Engl. & Prantl, Nat. Pflanzenfam., ed. 2, 17a(2): 99 (1959); Y. Tsiang & P. T. Li in Fl. Reip. Popul. Sin. 30(2): 39, t. 16(1–8) (1979); Fl. Guizhou 1: 271, fig. 237 (1982); P. T. Li in Fl. Guangdong 2: 15, t. 11(1–5) (1987); P. T. Li in Fl. Guangxi 1: 122 (1991); S. H. Yuan in Fl. Yunnan 5: 15, t. 4(1–9) (1991); P. T. Li & Z. K. Li in High. Pl. China 3: 166, t. 255(1–8) (2000); N. T. Ban, Fl. Viet. 1: 307 (2000); P. T. Li & M. G. Gilbert in Fl. China 19: 680 (2011). Type: China. Province du Kouy-Tchéou: fourrés près du Houa-Kiang, 6 June 1904, J. Cavalerie & J. J. Fortunat 2051 (holotype: P! [P00160893]; isotypes: E! [E00092498, E00092500], K! [K001089909, K001089910], P! [P00432372, P00432373]).
Euodia lyi H. Lév., Bull. Acad. Int. Geogr. Bot. 24 (294): 142–143 (1914), et Fl. Kouy-Tcheou: 376 (1915), as ‘Evodia’. Type: China. Kouy-Tchéou: Juin Lin 1912, *J. Cavalerie 3971* (lectotype: E! [E00092504], designated by Turner (2018); isolectotypes: A! [A00096362], P! [P00160849]).
Miliusa balansae auct. non Finet & Gagnep., Chaowasku & Kessler, Nordic J. Bot. 32: 301 (2014), pro parte.

##### Chinese name.

中华野独活 Zhōng Huá Yě Dú Huó.

##### Description.

Shrubs or small trees, 2–6 m tall, up to 26 cm in diam. Annual branches densely covered with spreading grayish yellow hairs ca. 0.5 mm long, 1–1.5 mm in diam., indumentum gradually deciduous; older branches sparsely pubescent or glabrous, without lenticels, longitudinally striate. Petiole 2–3 × 1.5 mm, indumentum as on branchlets; leaf blades thinly chartaceous, elliptic or ovate-elliptic, rarely obovate-elliptic or narrowly elliptic, 5–15 × 2–5.5 cm; apex acuminate or obtusely acute, tip 5–20 mm; base rounded or obtuse, sometimes slightly asymmetric; adaxial surface glabrous except for the pubescent midvein, midvein slightly impressed, lateral veins inconspicuous; abaxial surface densely covered with ascending hairs ca. 0.5 mm long; lateral veins ca. 9–12 per side, arched-ascending at 60°–85° from the midvein, anastomosing ca. 5–7 mm from the margin. Inflorescences axillary, 1(–2)-flowered; flowers bisexual, buds yellowish green, turning red at anthesis, ca. 1 cm in diam.; peduncle ca. 1 mm long, with 1–2 bracts, ovate, 0.5–1 × 0.5 mm; pedicel slender, 35–75 × 0.5 mm, densely covered with spreading hairs ca. 0.5 mm long; bracteoles 1–2, ovate, 1 × 0.5 mm. Sepals 3, free, ovate-triangular, 2–3 × 1.5–2 mm, densely covered with spreading yellow hairs ca. 0.3 mm long. Petals 6, in 2 whorls of 3; outer petals free, narrowly ovate-triangular, 3–4 × 1 mm, densely covered with spreading yellow hairs ca. 0.3 mm long; inner petals ovate, 10–18 × 7–10 mm, abaxially sparsely pubescent, margins appressed from base to about the midpoint, upper 1/3–1/2 reflexed, lower half saccate, with 5–7 veins and nearly translucent “windows” between veins, with conspicuous red tuberculate protuberances along the veins. Stamens in ca. 7 whorls, 50–60, obovate, 1 × 1 mm; connective apex obtusely apiculate. Carpels 45–60, ca. 1.5 mm long; ovary ovate, ca. 1 mm long, sparsely pubescent; stigma narrowly ovate, ca. 0.5 mm long, minutely pubescent; ovules 2(–3) per carpel. Torus cylindrical, densely white-pubescent. Fruiting peduncle 2 × 1.5–2 mm; fruiting pedicel 50–90 × 1.5 mm. Monocarps 15–30, ellipsoid-globose, 9–14 × 8–10 mm, glabrous, finely tuberculate, slightly constricted at the middle, apex mucronate with a tip ca. 0.3 mm long; stipes 12–21 × 0.8 mm, densely short-pubescent. Seeds 1–2 per monocarp, ellipsoid, slightly flattened, 7 × 5 mm.

##### Phenology.

Flowering from March to September; fruiting from May to December.

##### Distribution.

Distributed in southern China (Guangdong: Yangchun; Guangxi: Baise, Da’e, Daxin, Debao, Donglan, Fengshan, Huanjiang, Jingxi, Leye, Lingle, Lingyun, Long’an, Longjin, Longlin, Longzhou, Nandan, Napo, Ningming, Pingguo, Rong’an, Tian’e, Tianlin, Tianyang, Xilin, Xincheng, Youjiang, Zhenfeng; Guizhou: Anlong, Anshun, Ceheng, Chishui, Libo, Luodian, Wangmo, Xingren, Xingyi, Xiuwen; Yunnan: Funing, Guangnan, Shizong, Xichou, Yuanjiang) and extending into Vietnam; also cultivated in the Xishuangbanna Botanical Garden (Yunnan).

##### Habitat.

Dense forests on limestone mountains and valley thickets at elevations of 600–2100 m.

##### Notes.

This species is widely distributed in Guangxi and parts of Yunnan and Guizhou but has not been recorded from Guangdong or Hainan. Its range is considerably narrower than that of *Miliusa balansae*; however, it extends further north, reaching Chishui City in Guizhou Province (28.405°N). The species predominantly occupies limestone montane habitats and shows greater light demand and drought tolerance, suggesting substantial ecological differentiation between the two species.

Based on similarity in floral and fruit characters, Chaowasku and Kessler (2014) reduced *Miliusa sinensis* to *M. balansae*, a treatment subsequently followed by Turner (2018). Based on our current data, we are unable to support this taxonomic interpretation. Detailed examination of extensive herbarium material, together with field observations of wild populations, reveals several stable and well-marked differences between *Miliusa sinensis* and *M. balansae*: (1) sepals of *M. sinensis* ovate-triangular, 2–3 × 1 mm, versus broadly ovate-triangular, 2–2.5 × 1.5–2 mm, in *M. balansae*; (2) outer petals of *M. sinensis* narrowly ovate-triangular, 3–4 × 1 mm, versus ovate-triangular, 2.5–3 × 1.5 mm, in *M. balansae*; (3) inner petals of *M. sinensis* smaller, usually 10–18 mm long, versus larger inner petals, usually 15–25 mm long, in *M. balansae*; (4) monocarps of *M. sinensis* smaller, usually 9–14 × 8–10 mm, versus larger monocarps, usually 12–22 × 10–14 mm, in *M. balansae*; and (5) branchlets, leaves, and pedicels of *M. sinensis* usually with spreading pubescence (hairs ca. 0.5 mm long), versus shorter puberulent (hairs 0.1–0.2 mm long) or sometimes glabrous in *M. balansae* (Table 1, Figs 6, 7).

##### Selected specimens examined.

**Guangxi** • **Baise City**, 11 Aug 2007, S. J. Fang & P. Y. Lin et al. 07LN489 (SZG); **Da’e County**, 1957, Z. Huang 43252 (IBSC, IBK, KUN); • **Daxin County**, 27 Mar 1960, Z. D. Gui 109 (IBK); • **Debao County**, 04 2010, Y. F. Huang & J. Huang 21664 (GXMG); • **Donglan County**, 4 Nov 1979, Q. H. Lv 5101 (IBK); • **Fengshan County**, 2012, X. Y. Huang & Y. D. Peng et al. s.n. (GXMG); • **Huanjiang County**, 21 Oct 1991, Q. Dian 70090 (KUN); • **Jingxi County**, 5 Nov 2020, P. Yang & S. Y. Nong et al. BL1398 (IBK); • **Leye County**, 17 Jun 2018, X. H. Hu & B. Pan et al. GXIBHXH003B04 (KUN); • **Lingle County**, 3 Jul 1959, Z. T. Li 603548 (IBSC, IBK); • **Lingyun County**, 3 Jun 1960, C. F. Liang 34093 (IBK); • **Long’an County**, 10 Nov 2011, J. C. Yang & Y. B. Liao LH1205 (IBK); • **Longjin County**, 5 Oct 1958, H. Q. Li 40088 (KUN); • **Longzhou County**, 7 Oct 1979, G. Nong 10939 (IBK); • **Nandan County**, 17 Apr 2010, J. Huang & X. M. Luo et al. 22036 (GXMG); • **Napo County**, 19 Mar 2013, Z. Z. Lan & Y. Li et al. 451026130319045LY (GXMG); • **Ningming County**, 14 Mar 2010, Y. D. Peng & Y. F. Huang et al. 21772 (GXMG); • **Pingguo County**, 2015, H. Z. Lv & B. Y. Huang et al. s.n. (GXMG); • **Rong’an County**, 29 Nov 2008, N. F. Li & L. H. Gao 19317 (GXMG); • **Tian’e County**, 9 Sept 1989, J. D. Bei 896923 (PE); • **Tianlin County**, 2 Jun 2013, L. Tian 451029130602033 (GXMG, GXMI); • **Tianyang County**, 2015, Y. Tian s.n. (GXMG); • **Xilin County**, 2014, Y. M. Zhao & N. F. Li et al. s.n. (GXMG); • **Xincheng County**, 11 Nov 1991, W. J. Zhang et al. 91088 (KUN); • **Youjiang District**, 2012, Youjiang Team s.n. (GXMG); • **Zhenfeng County**, 12 Sept 1936, S. W. Deng 90833 (IBK). **Guizhou • Anlong County**, 11 Jul 1959, S. D. An 74 (HGAS, PE); • **Anshun County**, 1990, K. M. Lan 900488 (GZAC); • **Ceheng County**, 4 Sept 1958, Z. Y. Cao 537 (HGAS, PE); • **Chishui City**, 13 May 2017, M. C. Wang 520402170513317LY (GZTM); • **Libo County**, 1 Nov 2018, J. W. Yang & X. F. Li et al. GZBG036B06 (KUN); • **Luodian County**, 3 May 1991, Q. Dian 40087 (KUN); • **Wangmo County**, 12 Sept 1936, S. W. Deng 90833 (SN); • **Xingren County**, 10 Jul 1960, Z. S. Zhang & Y. T. Zhang 5966 (HGAS); • **Xingyi City**, 19 Aug 1929, B. Q. Zhong 1690 (PE); • **Xiuwen County**, 24 Jun 1996, 94-senbao s.n. (GFS); • **Zhenfeng County**, 12 Sept 1936, S. W. Deng 90833 (IBSC). **Yunnan • Funing County**, 16 Aug 1961, S. G. Wu 3685 (HITBC, KUN); • **Guangnan County**, alt. 1200 m, 23.39°N, 105.29°E, 18 Oct 2002, H. Wang 6516 (HITBC, PE); • **Mengla County**, 29 Jun 2017, C. Y. Sheng C400787 (HITBC); • **Shizong County**, 12 Jul 2020, E. D. Liu & C. H. Wang LED9730 (KUN); • **Xichou County**, 2 Jun 1964, S. Z. Wang 608 (KUN); • **Yuanjiang County**, 7 Jun 1984, G. D. Tao 38853 (HITBC, KUN). The complete list of specimens is provided in Suppl. material 1.

#### 7. Miliusa xiaoboi

Taxon classificationPlantaeMagnolialesAnnonaceae

X.L.Hou & Z.Y.Shi sp. nov.

79E8E7DA-0265-510E-A091-80801462A55F

urn:lsid:ipni.org:names:77378909-1

Fig. 8

##### Chinese name.

肖波野独活 Xiāo Bō Yě Dú Huó.

**Figure 8.**
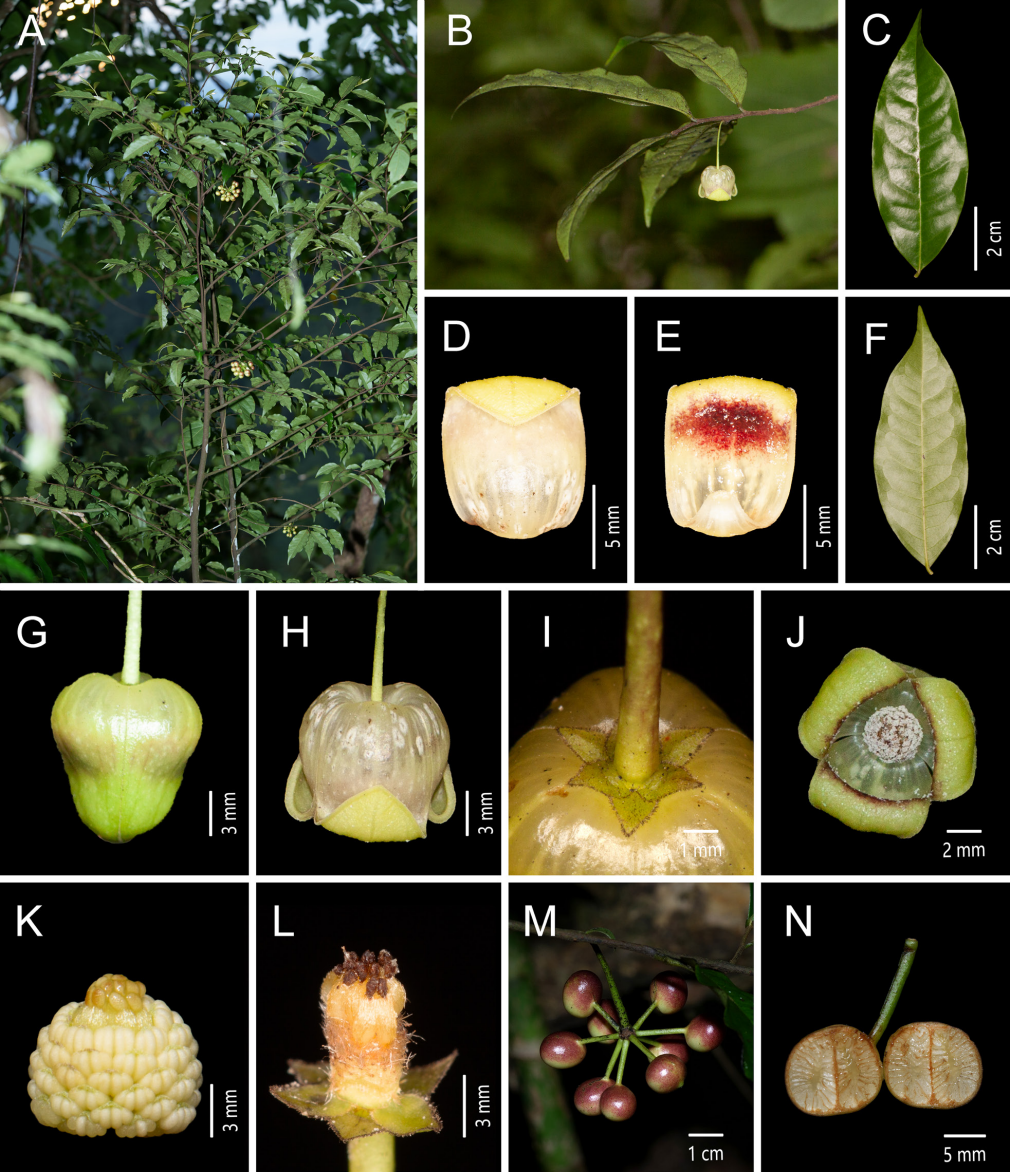
*Miliusa xiaoboi* X. L Hou & Z. Y. Shi. **A**. Plant; **B**. Flowering branch; **C**. Adaxial and abaxial view of the leaf blade; **D**. Abaxial view of the inner petal; **E**. Adaxial view of the inner petal; **F**. Abaxial view of the leaf blade; **G**. Lateral view of the flower bud; **H**. Lateral view of the flower; **I**. Bottom view of the flower (showing the sepals and inner petals); **J**. Bottom view of the flower; **K**. Androecium and gynoecium; **L**. Flower without inner petals and androecium; **M**. Fruiting branch; **N**. Longitudinal section of fruiting [photos: **A, M, N** by X. L. Hou **B–L** by Z. Y. Shi].

##### Type material.

**China** • **Yunnan Province**: Yakouzai, Xinjie Town, Wenshan City, alt. 1450 m, 23.151569, 103.977406, 9 Jan 2025, *Z. Y. Shi 2025010901* (holotype: AU; isotype: AU).

##### Diagnosis.

This species resembles *Miliusa balansae* but can be readily distinguished by its yellow flowers (vs. red), by its pedicels 1.9–3.3 cm long (vs. 4–6.5 cm), and by its stipes 6–12 mm long (vs. 15–30 mm).

##### Description.

Small tree, up to 5 m tall. Annual branches ca. 1 mm in diam., densely covered with appressed grayish yellow hairs ca. 0.3 mm long; older branches gradually glabrescent, longitudinally striate. Petiole 2–3 mm long, adaxially grooved, abaxially transversely wrinkled, slightly swollen, grayish yellow pubescent; leaf blades thinly chartaceous, elliptic or ovate-elliptic, rarely narrowly elliptic or obovate-elliptic, (6–)8–13 × (2.2–)2.8–4.5 cm, apex acuminate with an acumen 0.5–2 cm long, base broadly cuneate to obtuse, slightly asymmetrical, margin slightly undulate; midvein adaxially impressed, abaxially raised; leaf blade between lateral veins slightly raised adaxially; lateral veins adaxially inconspicuous, abaxially slightly raised, 9–12 per side, arched-ascending at 55°–65° from the midvein, anastomosing 4–5 mm from the margin; intersecondary veins well developed, 5–7 per side; tertiary veins forming a conspicuous reticulation; adaxial surface dark green, glabrous except for the grayish yellow pubescent midvein; abaxial surface pale green, slightly grayish white, sparsely white-pubescent to glabrescent, with dense transparent glandular dots. Inflorescences axillary on young or leafless older branches, 1-flowered; flowers bisexual; peduncle less than 1 mm long, with 2 bracts, ovate, 1.5 × 1 mm, sparsely puberulent; flower buds yellowish green, conical, slightly constricted medially, ca. 1 cm in diam. at anthesis; pedicel 19–30 mm long, ca. 1 mm in diam. at the middle, gradually thickening distally, clavate, green to pale red in vivo, nearly glabrous, with a single basal bracteole, ovate, 2 × 0.8 mm, sparsely puberulent. Sepals 3, free, ovate, 1.5 × 1.2 mm, apex shortly acuminate, abaxially densely puberulent with hairs ca. 0.2 mm long, ciliate, adaxially glabrous. Petals 6, in 2 whorls of 3; outer petals similar to sepals; inner petals ovate, 16–18 × 9–10 mm, margins appressed from base to ca. 3/4 of the length, upper 1/4 reflexed, yellow, both surfaces white-puberulent, apex obtuse, base saccate, yellowish white, with 7–9 veins and distinct translucent “windows” between veins; adaxial surface with a purplish red, transversely elliptic patch ca. 8 × 3 mm in the median region, accompanied by tuberculate protuberances. Stamens in ca. 7 whorls, 60–70, 1 × 0.9 mm; connective pale green in vivo, apex obtuse with a tip ca. 0.1 mm long; anthers white, oblong, ca. 0.6 mm long; filaments ca. 0.3 mm long. Carpels 15–21, ca. 1.5 mm long; ovary oblong, ca. 1 mm long, densely white-pubescent with hairs ca. 1 mm long; stigma ellipsoid, ca. 0.5 mm long, glabrous; ovules 2 per carpel. Torus cylindrical, ca. 1.5 mm high, densely white villous, hairs ca. 1 mm long. Fruiting peduncle 1–2 × 1–1.5 mm; fruiting pedicel 25–35 mm long, 1–1.2 mm in diam. at the middle, gradually thickening distally, glabrous. Stipes 6–12 × 0.8 mm, glabrous. Monocarps red to purplish black at maturity, ellipsoid, berry-like, 11–13 × 9–11 mm, smooth in vivo, 9–12 × 8–10 mm when dry, often obliquely attached to the seed-bearing portion, finely tuberculate, apex with an inconspicuous tip. Seeds 1–2 per monocarp, hemispherical, 7 × 5 mm, often with a median constriction.

##### Phenology.

Flowering from September to January (extending into the following year); fruiting from April to September.

##### Distribution.

Currently known only from Yunnan, China (Funing, Jinghong, Malipo, Mengla, Ruili, Wenshan, Xichou).

##### Habitat.

Damp, dense forests on limestone mountains at 850–1450 m.

##### Etymology.

The specific epithet honors Bo Xiao, a forestry technologist who assisted in collecting material for this study.

##### Selected specimens examined.

**Yunnan • Funing County**, 11 Oct 1965, 65-Wenshan Team 079 (KUN, PE); • Maguan County, Renhe Township, alt. 1545 m, 22.91801186°N, 104.2947686°E, 2 Dec 2025, B. Xiao 2025120201 (AU); • Jinghong City, 5 Dec 1993, H. Wang & B. G. Li 110 (HITBC); • **Malipo County**, alt. 1416 m, 23.4913862°N, 105.1654296°E, 17 Dec 2019, E. D. Liu & J. Zou LED9379 (KUN); • ibid, 3 Jan 1940, C. W. Wang 86106 (NF, PE); • ibid, alt. 1500 m, 22 Nov 1947, K. M. Feng 13549 (PE); • ibid, 27 Oct 2015, Malipo Expedition Team 5326240595 (HITBC); • **Mengla County**, alt. 850 m, 5 Sept 2012, J. W. Li 1830 (HITBC); • ibid, alt. 1000 m, Oct 1936, C. W. Wang 79984 (IBSC, KUN, PE); • ibid, alt. 650m, 21.5°N, 101.21°E, 31 Jan 1978, P. Q. Wang 11113 (HITBC); • ibid, alt. 700m, 21.54°N, 101.13°E, 12 May 1973, Y. H. Li 8482 (HITBC); • ibid, 7 Mar 1946, X. H. Li 96029 (NAS); • **Jinghong City**, alt. 1430 m, 10 Dec 1961, T. H. Li 3816 (HITBC, IBK, IBSC, KUN); • ibid, alt. 1300 m, 6 Dec 1993, H. Wang & B. G. Li 3031 (PE); • **Ruili City**, alt. 994 m, 24.1189°N, 97.9806°E, 15 Apr 2017, X. L. Hou & R. P. Zhang 90699 (AU); • ibid, alt. m, 24.1818601°N, 98.0128286°E, 10 Dec 2020, E. D. Liu & Z. W. Liu et al. LED10316 (KUN); • **Wenshan County**, 17 Oct 2019, Y. S. Chen WS0376 (IBSC); • ibid, Yakouzai, Xinjie Town, alt. 1450 m, 23.1515693°N, 103.9774061°E, 27 Jul 2025, Y. J. Li 383, 390 (AU).

#### 8. Miliusa dioeca

Taxon classificationPlantaeMagnolialesAnnonaceae

(Roxb.) Chaowasku & Kessler, Willdenowia 43: 104 (2013)

EF260ABA-F030-58AD-A19A-87CB00EDE254

Figs 9, 10

Miliusa dioeca (Roxb.) Chaowasku & Kessler, Willdenowia 43: 104 (2013); H. B. Ding et al., Biodivers. Sci. 31: 23254 (2023). –– Uvaria dioeca> Roxb., Fl. Ind. 2: 659 (1832). –– Phaeanthus dioecus (Roxb.) Kurz, J. Asiat. Soc. Bengal, Pt. 2, Nat. Hist. 39: 62 (1870), as ‘dioicus’. Type: Roxburgh s.n. (lectotype: BM! [BM000595529], designated by Chaowasku (2013)).
Miliusa tenuistipitata W. T. Wang in Acta Phytotax. Sin. 6: 200 (1957); Y. Tsiang et P. T. Li in Fl. Reip. Popul. Sin. 30 (2): 40, t. 16 (13–15) (1979); S. H. Yuan in Fl. Yunnan. 5: 16, t. 4 (10–14) (1991); P. T. Li et Z. K. Li in High. Pl. China 3: 166, t. 255 (9–11) (2000); P. T. Li & M. G. Gilbert in Fl. China 19: 679 (2011). Type: Yunnan: lan-tsang, alt. 1500 m, in silvia mixtra, 6 m alta, fl. viridis, May 1936, *C. W. Wang 76517* (holotype: PE! [PE00028367]; isotypes: A! [A00039452], PE! [00934534]). syn. nov.

##### Chinese name.

云南野独活 Yún Nán Yě Dú Huó.

**Figure 9.**
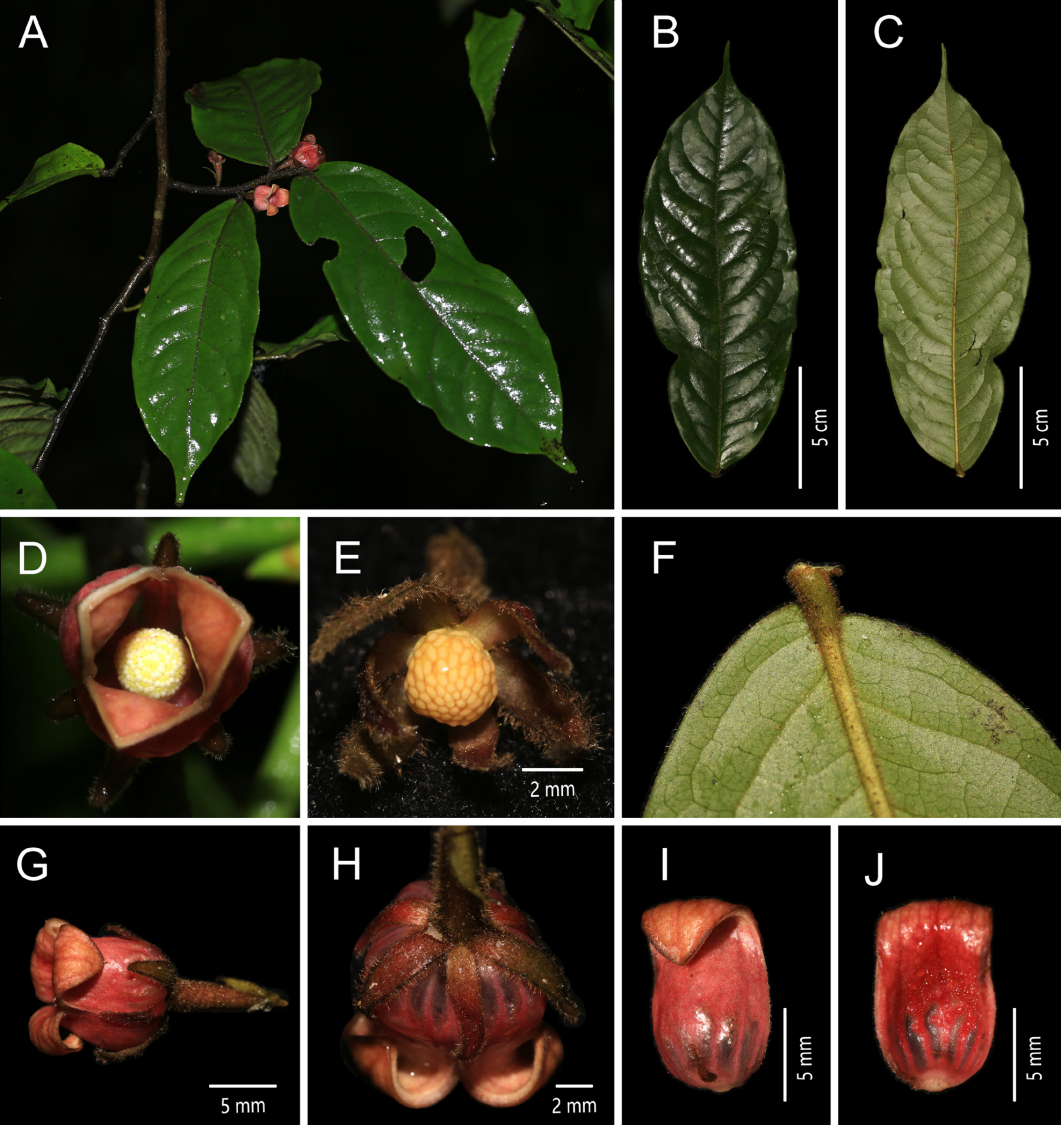
*Miliusa dioeca* (Roxb.) Chaowasku & Kessler. **A**. Flowering branch; **B**. Adaxial view of the leaf blade; **C**. Abaxial view of the leaf blade; **D**. Male flower; **E**. Female flower; **F**. The base of leaf blade (showing asymmetrical base); **G**. Lateral view of the flower; **H**. Bottom view of the flower; **I**. Abaxial view of the inner petal; **J**. Adaxial view of the inner petal [photos: **A–J** by X. L. Hou].

**Figure 10.**
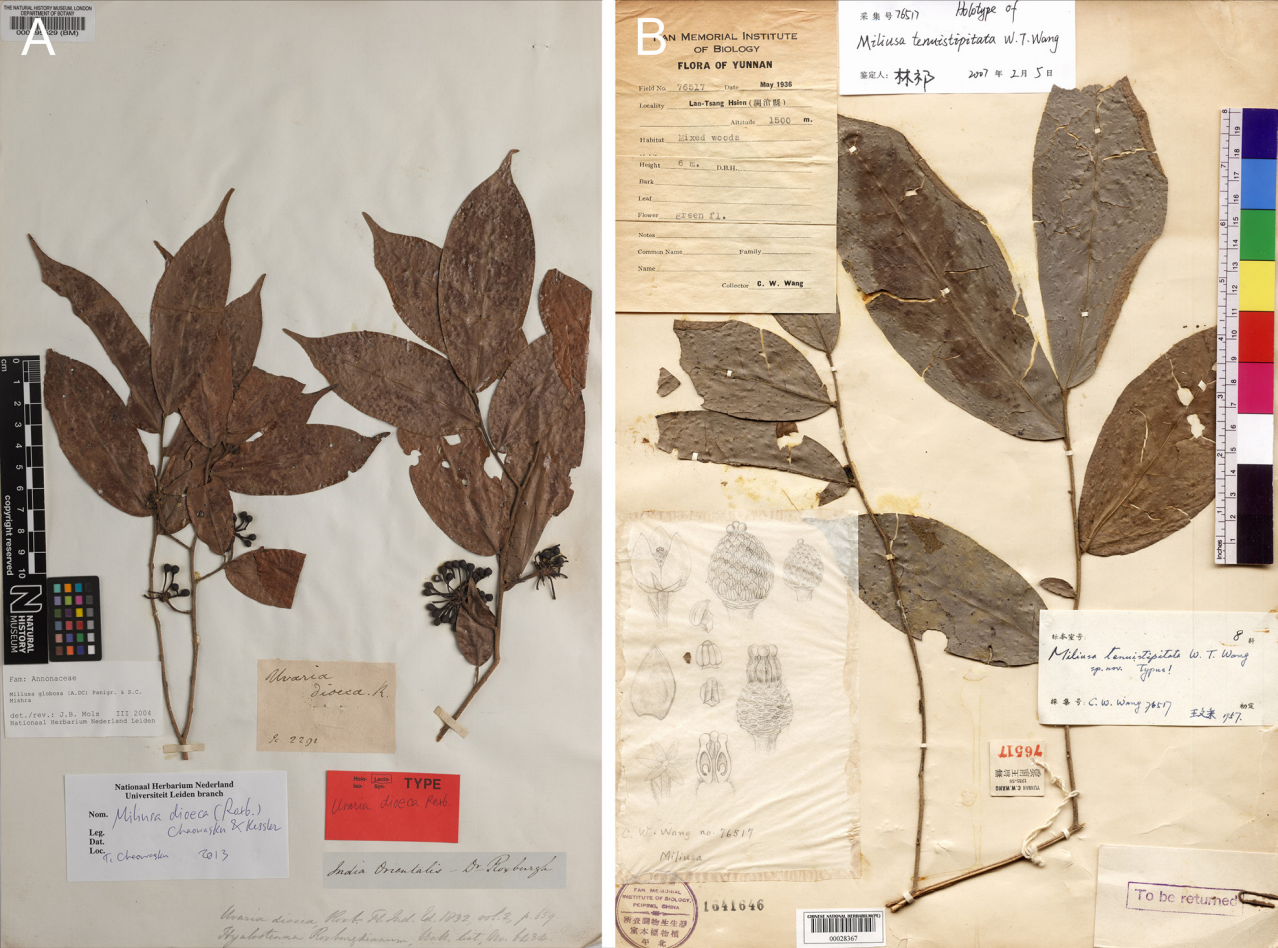
Comparison the type specimens of *M. dioeca* (Roxb.) Chaowasku & Kessler and *M. tenuistipitata* W. T. Wang. **A**. Lectotype of *M. dioeca* (BM [BM000595529]); **B**. Holotype of *M. tenuistipitata* (PE [PE00028367]).

##### Description.

Shrubs or small trees, 2–12 m tall, up to 30 cm in diam. Annual branches ca. 2 mm in diam., covered with spreading yellowish gray hairs, gradually glabrescent; older branches yellowish brown, glabrous, without lenticels. Petiole 2–4 × 2 mm, densely yellowish gray pubescent; leaf blades chartaceous or membranous, oblong or slightly obovate-oblong, 8–19 × 3–7 cm; apex acuminate or shortly acuminate; base rounded or broadly cuneate, often asymmetrical; adaxial surface glabrous except along the slightly impressed midvein; abaxial surface with ascending short hairs; lateral veins 8–13 per side, arched-ascending at 45°–65° from the midvein, anastomosing ca. 7–9 mm from the margin; tertiary veins conspicuous or obscure. Inflorescences axillary, 1–2-flowered; flowers bisexual or unisexual, similar in morphology, purplish red; female flowers with several reduced stamens, male flowers without rudimentary pistils; peduncle 1 mm long, with 1 bract, ovate, 2 × 1 mm; pedicel 10–20(–30) mm long, ca. 0.5 mm in diam. at the middle, slightly thickened upwards, clavate, densely covered with spreading yellowish gray hairs; with 1 bracteole on the lower half, narrowly ovate, 3 × 1 mm, sometimes leaf-like, densely pubescent. Sepals 3, free, narrowly ovate, 4–8 × 1.5–2 mm, densely pubescent. Petals 6, in 2 whorls of 3; outer petals similar to sepals but slightly smaller, 4–7 × 1.5–2 mm; inner petals ovate, 10–15 × 8–11 mm, margins appressed from base to ca. 2/3 of the length, upper 1/3 reflexed, apex acute, saccate below the middle, with 5 veins and nearly translucent “windows” between veins, sparsely pubescent outside, nearly glabrous inside. Stamens in 5–7 whorls, 50–70, 1 × 1 mm; connectives apiculate at apex. Carpels 20–30, ca. 1.5 mm long; ovary sparsely short-pubescent; stigma ellipsoid, glabrous; ovule 1, basal. Torus cylindrical, pubescent. Fruiting peduncle 2 × 2.5 mm; fruiting pedicel 12–25(–35) × 2–2.5 mm, pubescent. Monocarps 10–25, ellipsoid, 12–14 × 8 mm, glabrous, densely and finely tuberculate; stipes 13–18 × 1 mm, glabrous.

##### Phenology.

Flowering from February to May; fruiting from May to August.

##### Distribution.

Occurs in China, in Xizang (Motuo) and Yunnan (Cangyuan, Jinghong, Lancang, Menghai, Menglian, Ningjiang, Simao, and Yingjiang), and extends west and south to India, Nepal, Bhutan, Bangladesh, and Myanmar.

##### Habitat.

Mixed forests and valley thickets at 350–1500 m.

##### Notes.

Phylogenetic analyses recovered *Miliusa dioeca* (India) nested within two accessions of *M. tenuistipitata* from China, forming a strongly supported clade (Fig. 1; BS ≥ 98, PP = 1.00). *Miliusa tenuistipitata* closely resembles *M. dioeca* in twig, leaf, flower, fruit, and indumentum characters. Detailed examination of the type material (Fig. 10) and additional specimens confirmed that these names apply to a single species. Because *Miliusa dioeca* was published earlier than *M. tenuistipitata*, we treat *M. tenuistipitata* as a synonym of *M. dioeca*.

##### Selected specimens examined.

**Xizang • Motuo County**, alt. 700 m, 23 Jul 1980, T. Sheng 11249 (PE). **Yunnan • Cangyuan County**, 2 Nov 1989, G. D. Tao & X. W. Li 10097 (KUN); • ibid, 2 Nov 1989, G. D. Tao & X. W. Li 40097 (HITBC); • ibid, 5 Jul 1974, Y. H. Li 12585 (HITBC, KUN); • ibid, 19 Jun 1974, T. H. Li 12355 (HITBC, KUN); • ibid, 19 Mar 1976, Y. H. Li 20095 (HITBC); • **Fohai County**, alt. 1300 m, Jun 1936, C. W. Wang 74832 (IBSC, KUN); • **Jinghong City**, Mar 1990, H. Wang 1914 (HITBC); • ibid, 3 Jan 1990, H. Wang 1910 (HITBC); • ibid, 3 Jan 1990, H. Wang 1916 (HITBC); • ibid, 3 Jan 1990, H. Wang 1928 (HITBC); • ibid, 7 Nov 1988, G. D. Tao 44882 (HITBC); • ibid, 7 Nov 1988, G. D. Tao et al. 44882 (HITBC); • ibid, 30 Jan 1990, H. Wang 1924 (HITBC); • **Lancang County**, alt. 1500 m, May 1936, C. W. Wang 76517 (PE); • **Menghai County**, alt. 1150 m, 12 Dec 1958, W. C. Wang 10326 (KUN, PE); • ibid, alt. 1300 m, Jun 1936, C. W. Wang 74832 (PE); • **Menglian County**, 30 Oct 2011, X. L. Hou 11103002 (AU); • ibid, 30 Oct 2011, X. L. Hou 11103003 (AU); • **Ningjiang County**, 6 Dec 1951, G. M. Feng 14185 (KUN); • ibid, alt. 800 m, 6 Dec 1951, G. M. Feng 14165 (PE); • **Simao City**, 26 Aug 1984, 234 (KUN); • ibid, alt. 700 m, 20 Apr 2000, H. Wang 4210 (HITBC, KUN); • **Yingjiang County**, 2 Apr 1979, Anonymous 06–103 (HITBC); • ibid, alt. 401 m, 24.77°N, 97.5794°E, 16 Apr 2017, X. L. Hou & R. P. Zhang 90712 (AU); • ibid, alt. 401 m, 24.77°N, 97.5794°E, 16 Apr 2017, X. L. Hou & R. P. Zhang 90715 (AU); • ibid, alt. 401 m, 24.77°N, 97.5794°E, 16 Apr 2017, X. L. Hou & R. P. Zhang 90716 (AU); • ibid, alt. 397 m, 24.77126°N, 97.59167°E, 20230321, J. W. Li et al. WPY932 (PE).

#### 9. Miliusa cuneata

Taxon classificationPlantaeMagnolialesAnnonaceae

Craib in Bull. Misc. Inform. Kew 1912: 145 (1912)

A4EBE451-E8A8-5C71-8087-5B519C0096A3

Fig. 11

Miliusa cuneata Craib in Bull. Misc. Inform. Kew 1912: 145 (1912); S. H. Yuan in Fl. Yunnan 5: 18, t. 4(5–19) (1991); P. T. Li & M. G. Gilbert in Fl. China 19: 679 (2011), excl. syn. Miliusa bannaensis; Fl. Thailand 16(1): 171 (2022). Type: Thailand. Chiang Mai, Doi Sutep, 14 May 1911, A. F. G. Kerr 1837 (lectotype: K! [K001089914], designated by Turner (2018); isolectotypes: ABD, B! [B100591459], BK, BM! [BM000547335], E! [E00092598], K! [K001089913], TCD! [TCD0009895], UC).
Miliusa elongata Craib, Bull. Misc. Inform. Kew 1925 (1): 12 (1925). Type: Thailand, Nakawn Sawan, Klawng Kung, 2 June 1922, A. F. G. Kerr 6050 (lectotype: K! [K001089919], designated by Kessler et al. (1995); isolectotypes: ABD, B! [B100591456], BK! [BK257663], BM! [BM000547336], E! [E00092595], P! [P00411063], TCD! [TCD0009893], UC).

##### Chinese name.

楔叶野独活 Xiē Yè Yě Dú Huó.

**Figure 11.**
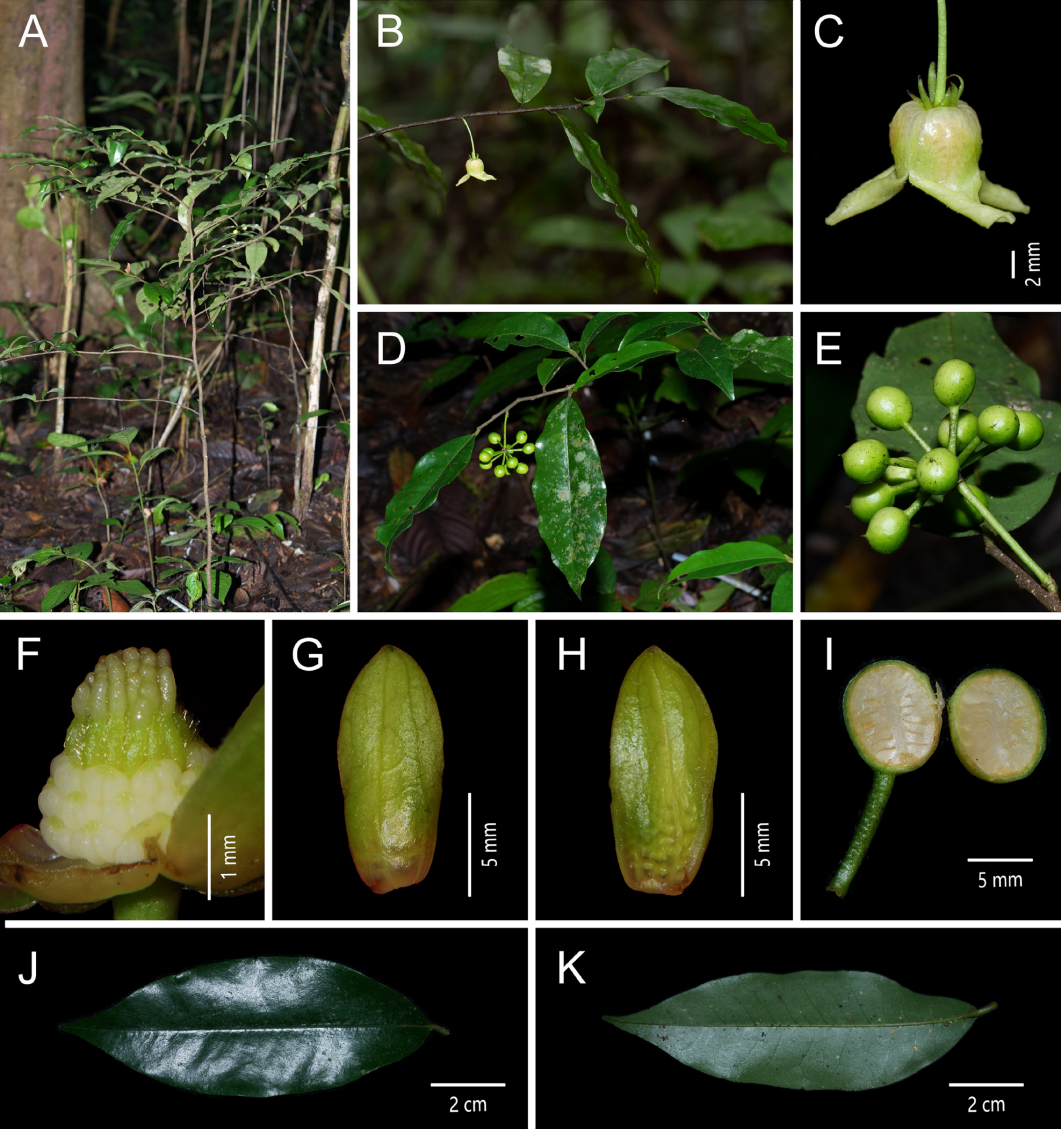
*Miliusa cuneata* Craib. **A**. Plant; **B**. Flowering branch; **C**. Lateral view of the flower; **D**. Fruiting branch; **E**. Fruit; **F**. Androecium and gynoecium; **G**. Abaxial view of the inner petal; **H**. Adaxial view of the inner petal; **I**. Longitudinal section of monocarp; **J**. Adaxial view of the leaf blade; **K**. Abaxial view of the leaf blade [photos: **A–K** by X. L. Hou].

##### Description.

Shrubs or small trees, 2–5 m tall. Branches 1.5–2 mm in diam., densely covered with ascending grayish yellow hairs, without lenticels. Petiole 3–4 × 1.5 mm, pubescent; leaf blades membranous, elliptic to oblong-lanceolate or obovate to obovate-lanceolate, 10–17 × 3–5.5 cm, apex acuminate with a tip 5–20 mm long, base cuneate or broadly cuneate, rarely subrounded; adaxial surface glabrous except for the shortly pubescent, impressed midvein, lateral veins slender and inconspicuous; abaxial surface initially with appressed short hairs, gradually glabrescent, midvein, lateral veins, and tertiary veins prominent; lateral veins 14–18 on each side, arched-ascending at 50°–65° from the midvein, anastomosing 5–7 mm from the margin. Inflorescences axillary, 1–3-flowered; flowers bisexual, yellow-green; peduncle 1–2 mm long, with 1–2 bracts, ovate, 1 × 0.5 mm; pedicel slender, 15–25 mm long, with 1 bracteole at the base, bracteole ovate, 1 × 0.5 mm. Sepals 3, ovate, 2–2.5 × 1–1.5 mm, apex acute, outer surface densely or sparsely pubescent, inner surface glabrous, reflexed at anthesis. Petals 6, in 2 whorls of 3; outer petals narrowly ovate-triangular, 3–4 × 1–1.5 mm, reflexed at anthesis; inner petals ovate-oblong, 12–15 × 6–8 mm, margins appressed from base to about the midpoint, upper half nearly horizontally spreading, margin revolute, lower half saccate, with 5–7 veins and transparent “windows” between veins near the base; inner surface with pale red-yellow patches along both sides of the midvein, turning black at maturity; outer surface glabrous, inner surface sparsely covered with spreading white hairs. Stamens in 4–5 whorls, 45–60, obovate, ca. 1 mm long; anther connective extended into a pointed tip. Carpels 20–35, ca. 2 mm long; ovary narrowly ovate, ca. 1.5 mm long, sparsely white-pubescent; stigma cylindrical, ca. 0.5 mm long, glabrous. Torus cylindrical, densely white-pubescent, hairs 0.8–1 mm long. Fruiting peduncle 2 × 1.5 mm; fruiting pedicel 20–35 × 1 mm, glabrous. Monocarps 8–20, ellipsoidal, slightly flattened, 8–9 × 6–7 mm, glabrous, densely covered with small tubercles, apex with a small pointed tip; stipes 7–10 × 0.5 mm, glabrous, often obliquely attached to the seed-bearing portion. Seed 1 per monocarp, ellipsoidal, 7 × 5 mm.

##### Phenology.

Flowering from February to July; fruiting from May to November.

##### Distribution.

Occurs in Yunnan, China (Jinghong, Mengla, Simao, Wanding, and Zhenyuan), and extends to Cambodia, Laos, Thailand, and Vietnam.

##### Habitat.

Moist valleys and montane forests at 500–1000 m.

##### Notes.

Yuan (1991) was the first to report *Miliusa cuneata* from China. Li and Gilbert (2011) accepted this treatment but erroneously placed *Miliusa bannaensis* in synonymy under *M. cuneata* (Xue and Tan 2016), thereby substantially broadening the species concept.

##### Selected specimens examined.

**Yunnan • Jinghong City**, 1956, S. Zhong 9536 (PE); • ibid, Jun 1991, H. Wang 56 (HITBC); • ibid, Jun 1991, H. Wang 58 (HITBC); • ibid, Oct 1991, H. Wang 96 (HITBC); • ibid, Sept 1936, C. W. Wang 78143 (PE); • ibid, Sept 1936, C. W. Wang 79141 (IBSC); • ibid, 3 Aug 1977, G. D. Tao et al. 16298 (HITBC); • ibid, 5 Sept 1992, H. Wang 76 (HITBC); • ibid, 14 Jul 1959, T. H. Li 1465 (KUN); • **Mengla County**, Nov 1982, C. D. Kao 34228 (HITBC); • ibid, alt. 580 m, 24 Jun 1988, Z. Y. Wu et al. 41 (KUN); • ibid, 3 Apr 1982, C. D. Kao 31667 (HITBC); • ibid, 6 Dec 1993, H. Wang & B. G. Li 107 (HITBC); • ibid, 1 Aug 2013, J. W. Li 3653 (HITBC); • **Simao City**, 25 Jul 2001, H. Wang 4843 (HITBC); • ibid, 26 Jul 2001, H. Wang 4846 (HITBC); • ibid, alt. 1000 m, 4 Oct 1955, P. Y. Mao 6481 (IBSC, KUN); • **Wanding City**, 19 Aug 1976, S. J. Pei 14050 (HITBC, KUN); • **Xishuangbanna Prefecture**, alt. 1000 m, 3 Jul 1978, X. R. Luo & Y. R. Lin 78159 (IBSC).

#### 10. Miliusa thorelii

Taxon classificationPlantaeMagnolialesAnnonaceae

Finet & Gagnep., Bull. Soc. Bot. France 54: 89–90, pl. III fig. E (1907)

B9A5B85A-1D98-5FF4-AEC8-6BBE1BE8B40F

Fig. 12

Miliusa thorelii Finet & Gagnep., Bull. Soc. Bot. France 54: 89–90, pl. III fig. E (1907); T. Chaowasku & P. J. A. Kessler in Nordic J. Bot. 31: 680–699 (2013); B. Xue & Y. H. Tan in Phytotaxa 282(2): 166–169 (2016); Fl. Thailand 16(1): 183 (2022). Type: Laos. Pak-lay, 1866–1868, C. Thorel 3301 (holotype: P! [P00160898]; isotypes: MPU! [MPU026918], P! [P00432370, P00432371], TCD! [TCD0009887, TCD0009888]).
Miliusa bannaensis X. L. Hou in Hou et al., Acta Phytotax. Sin. 42: 79, Fig. 1 (2004). Type: China. Yunnan: Mengla, Jingpiao, 800 m in humid forest, 10 December 1998, *H. Zhu & H. Wang 2125* (holotype: HITBC! [06285]).
Miliusa cuneata auct. non Craib, P. T. Li & M. G. Gilbert in Fl. China 19: 679 (2011), pro part.
Miliusa velutina auct. non J. D. Hooker & Thomson, P. T. Li & M. G. Gilbert in Fl. China 19: 680 (2011).

##### Chinese name.

版纳野独活 Bǎn Nà Yě Dú Huó.

**Figure 12.**
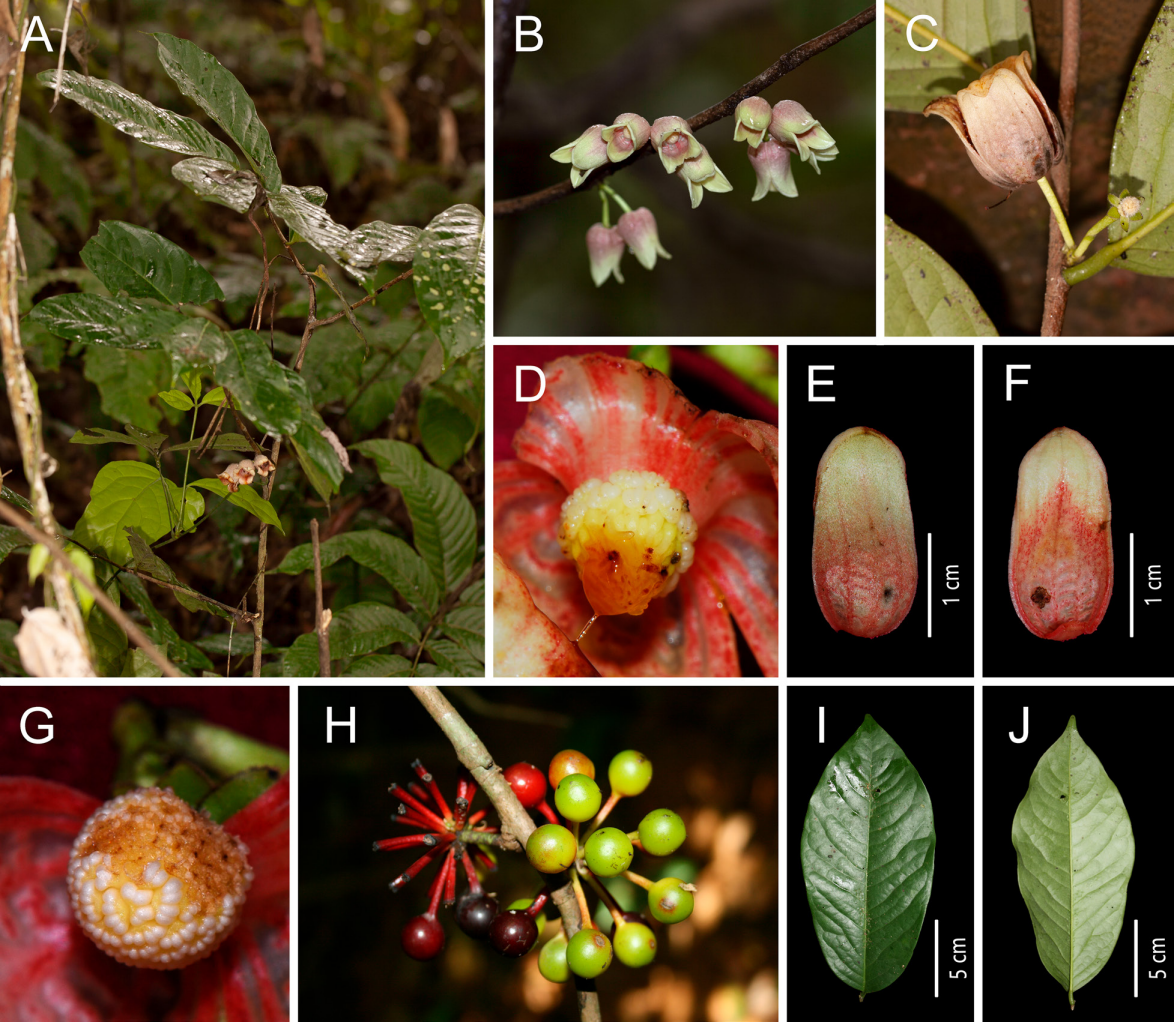
*Miliusa thorelii* Finet & Gagnep. **A**. Plant; **B**. Flowering branch; **C**. Lateral view of the flower; **D**. Bisexual flower; **E**. Abaxial view of the inner petal; **F**. Adaxial view of the inner petal; **G**. Male flower; **H**. Fruiting branch; **I**. Adaxial view of the leaf blade; **J**. Abaxial view of the leaf blade [photos: **A–J** by X. L. Hou].

##### Description.

Shrubs or small trees, 1.5–5 m tall. Annual branches glabrous, 2 mm in diam., gray-brown, with reticulate, sunken longitudinal striations. Petiole slightly swollen, 5–8 × 2.5–3 mm, shallowly grooved adaxially, smooth abaxially, glabrous, drying black; leaf blades chartaceous, glabrous, ovate-elliptic or obovate-elliptic, 14–26 × 6.5–10 cm, base rounded or slightly obtuse, asymmetric, apex acuminate and blunt-tipped, acumen 5–18 mm long, drying dark blue-green adaxially, pale brownish yellow abaxially; midvein nearly flat adaxially, prominent abaxially; lateral veins 13–15 per side, arched-ascending at 45°–60° from the midvein, anastomosing 7–10 mm from the margin, slightly raised adaxially, distinctly raised abaxially. Inflorescences axillary or on leafless older branches, 2–6-flowered; flowers bisexual or andromonoecious; peduncle 1–2 mm long; bracts 1–2, broadly ovate, 1.5–2 × 1.5–2 mm, grayish yellow pubescent; pedicel slender, ca. 10 mm long, 0.6 mm in diam., thickening toward the apex, with 1–2 bracteoles at the base and 1 bracteole at the mid-lower part; bracteoles broadly ovate, 1.5–2 × 1.5–2 mm, persistent in fruit. Sepals 3, free, broadly ovate, 1.5–2 × 1.5–2 mm, densely pubescent outside, glabrous inside. Petals 6, in 2 whorls of 3; outer petals ovate, 1.8–2 × 1.2–1.5 mm; inner petals pale purplish red, glabrous, ovate, ca. 20 × 13 mm, margins appressed from base to about the midpoint, upper 1/3 recurved, basal half saccate, with 5–7 veins and transparent “windows” between veins. Stamens in staminate flowers in ca. 9 whorls, 50–70 in number; in bisexual flowers 14–16, ca. 1.5 mm long; anther cells elliptic, connective extending beyond the anther cells to form a blunt tip. Carpels 25–45; ovary ellipsoid, ca. 3 mm long, sparsely white-pubescent; stigma cylindrical, 2 mm long. Torus narrowly ovoid, densely white-pubescent. Fruiting peduncle 2 × 3 mm; fruiting pedicel 10–17 × 2 mm; stipes 7–14 × 1 mm. Monocarps 8–30, subglobose, 8–9 mm in diam., glabrous, 1-seeded, drying black, wrinkled, pericarp thin, adhering to the seed coat.

##### Phenology.

Flowering from April to December; fruiting from June to February of the following year.

##### Distribution.

Occurs in Yunnan, China (Jiangcheng, Menghai, Mengla, and Xishuangbanna) and extends to Laos, Myanmar, and Thailand.

##### Habitat.

Sparse forests on montane and limestone terrain at 500–800 m.

##### Notes.

Li and Gilbert (2011) treated *Miliusa bannaensis* as a synonym of *M. cuneata*. However, clear differences in leaf-blade size and morphology and in flower color between the two taxa indicate that this synonymy is untenable. Xue and Tan (2016) followed Chaowasku and Kessler (2013b) in treating *Miliusa bannaensis* as a synonym of *M. thorelii*. They further stated that “the Chinese specimens identified as *Miliusa velutina* actually represent *M. thorelii*”, thereby excluding *M. velutina* from the flora of China (Xue and Tan 2016).

##### Selected specimens examined.

**Yunnan • Menghai County**, 9 Jun 2013, J. W. Li 3247 (HITBC, KUN); • ibid, 10 Aug 2014, J. W. Li 4140 (HITBC); • ibid, 25 May 2013, J. W. Li 3165 (HITBC, KUN); • **Mengla County**, 9 May 2006, Z. L. Bai c530109 (HITBC); • ibid, Jan 1989, H. Zhu & H. Wang 2416 (HITBC); • ibid, Jun 1991, H. Wang 63 (HITBC); • ibid, Jun 1991, H. Wang 69 (HITBC); • ibid, Jun 1991, H. Wang 94 (HITBC); • ibid, Jun 1992, H. Wang 55 (HITBC); • ibid, 2 Dec 1983, C. D. Kao 34868 (HITBC); • ibid, 3 Sept 1959, S. J. Pei 599766 (HITBC); • ibid, 5 Dec 1983, C. D. Kao 34965 (HITBC); • ibid, 12 Jul 1972, Y. H. Li 8459 (HITBC); • ibid, 12 Jun 2019, C. Y. Sheng C400854 (HITBC); • ibid, 23 May 2002, H. Wang 5671 (HITBC, PE); • ibid, 28 Apr 1977, G. D. Tao 9176 (HITBC); • ibid, alt. 700 m, 27 Dec 2001, X. L. Hou 0107 (IBSC); • ibid, alt. 750 m, 21.62523°N, 101.58645°E, 28 Apr 2011, X. L. Hou & T. X. Sun 11042806 (AU); • ibid, alt. 800 m, Nov 1936, C. W. Wang 80437 (PE); • **Xishuangbanna Prefecture**, 28 Oct 2008, X. L. Hou & T. X. Sun 8102805 (AU); • ibid, 28 Oct 2008, X. L. Hou & T. X. Sun 8102806 (AU); • ibid, alt. 800 m, 28 May 1980, K. S. Chow et al. 80036 (PE).

### Uncertain species

*Miliusa chantaburiana* was reported from Yingjiang, China, by Ding et al. (2023). However, detailed examination of the cited specimens revealed five major morphological discrepancies from the original description of the species by Damthongdee and Chaowasku (2018): (1) the stigma is cylindrical rather than horseshoe-shaped; (2) there are 1–2 ovules per ovary rather than a single ovule; (3) the midvein is raised adaxially rather than sunken; (4) the leaf base is broadly cuneate to nearly rounded rather than cuneate; and (5) there are 9–13 lateral veins per side rather than 7–10. In addition, molecular phylogenetic analysis based on specimen R. P. Zhang 90728 (AU) does not support a close relationship with *Miliusa chantaburiana* (Fig. 1). Further study is therefore required to clarify the identity of these Chinese collections.

#### Selected specimens examined.

**Yunnan • Yingjiang County**, Tongbiguan Nature Reserve, beside the Daying River, Taiping Town, alt. 591 m, 24.4484°N, 97.5969°E, 27 Jul 2022, S. S. Zhou et al. WPY151 (PE); • ibid, Mangyun Hongbeng River, alt. 299 m, 24.4376°N, 97.5300°E, J. W. Li 4668 (PE); • ibid, Tongbiguan Nature Reserve, Shiti Village, Taiping Town, alt. 541 m, 24.44787°N, 97.59593°E, 28 Jul 2022, P. Y. Wang et al. WPY57 (PE); • ibid, alt. 981 m, 24.46459°N, 97.61354°E, 18 Mar 2023, J. W. Li & H. B. Ding et al. WPY853 (PE); • ibid, alt. 944 m, 24.6677°N, 97.5991°E, 17 Apr 2017, X. L. Hou & R. P. Zhang 90728 (AU); • ibid, alt. 900 m, 27 Apr 1992, G. D. Tao 45495 (HITBC); • **Mengla County**, cultivated, alt. 550 m, 21.9266°N, 101.2533°E, 7 Apr 2017, X. L. Hou & R. P. Zhang 90560 (AU).

## Supplementary Material

XML Treatment for Miliusa

XML Treatment for Miliusa glochidioides

XML Treatment for Miliusa horsfieldii

XML Treatment for Miliusa aurilaveoides

XML Treatment for Miliusa longicarpa

XML Treatment for Miliusa balansae

XML Treatment for Miliusa sinensis

XML Treatment for Miliusa xiaoboi

XML Treatment for Miliusa dioeca

XML Treatment for Miliusa cuneata

XML Treatment for Miliusa thorelii

## Additional information

### Artificial Intelligence (AI) use

The authors accept full responsibility for the content of the manuscript, including the disclosure of any use of AI.

No AI tools were used in the preparation of this manuscript.

### Author ORCIDs

Z. Shi https://orcid.org/0000-0002-3995-1247

X. Hou https://orcid.org/0009-0001-2233-7486

### Data availability

All data that support the findings of this study are available in the main text and in Supplementary materials 1–4. Relevant molecular data can be found at GenBase and GenBank.

## Supplementary materials

Supplementary material 1 Specimen information Data type xlsx https://binary.pensoft.net/file/1601653This dataset is made available under the Open Database License (http://opendatacommons.org/licenses/odbl/1.0/). The Open Database License (ODbL) is a license agreement intended to allow users to freely share, modify, and use this Dataset while maintaining this same freedom for others, provided that the original source and author(s) are credited. Zhiyuan Shi, Xueliang Hou

Supplementary material 2 Database sequences Data type xlsx https://binary.pensoft.net/file/1601654This dataset is made available under the Open Database License (http://opendatacommons.org/licenses/odbl/1.0/). The Open Database License (ODbL) is a license agreement intended to allow users to freely share, modify, and use this Dataset while maintaining this same freedom for others, provided that the original source and author(s) are credited. Zhiyuan Shi, Xueliang Hou

Supplementary material 3 Maximum likelihood phylogenetic analysis of the *Miliusa* in China Data type pdf https://binary.pensoft.net/file/1601655This dataset is made available under the Open Database License (http://opendatacommons.org/licenses/odbl/1.0/). The Open Database License (ODbL) is a license agreement intended to allow users to freely share, modify, and use this Dataset while maintaining this same freedom for others, provided that the original source and author(s) are credited. Zhiyuan Shi, Xueliang Hou

Supplementary material 4 Bayesian phylogenetic analysis of the *Miliusa* in China Data type pdf https://binary.pensoft.net/file/1601656This dataset is made available under the Open Database License (http://opendatacommons.org/licenses/odbl/1.0/). The Open Database License (ODbL) is a license agreement intended to allow users to freely share, modify, and use this Dataset while maintaining this same freedom for others, provided that the original source and author(s) are credited. Zhiyuan Shi, Xueliang Hou
