# Supplementary figures and images for: A revision of the Miliusa (Annonaceae) from China
Source: PhytoKeys. 2026 Apr 21;273:185–223. doi: 10.3897/phytokeys.273.174592 (PMC13126048; doi:10.3897/phytokeys.273.174592)

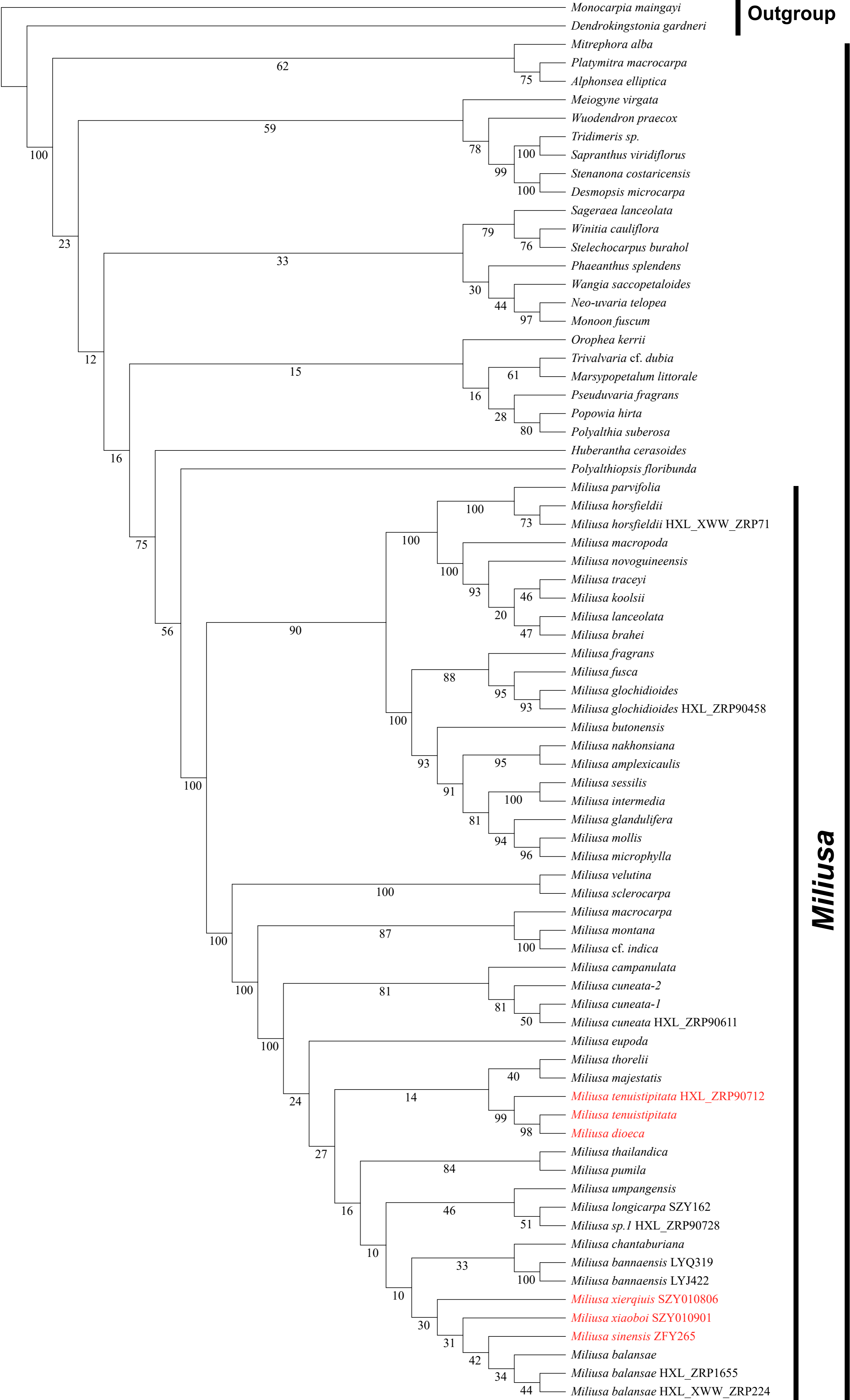

Supplement: Supplementary material 3 — Maximum likelihood phylogenetic analysis of the Miliusa in China [file phytokeys-273-185_article-174592__-s003.pdf]

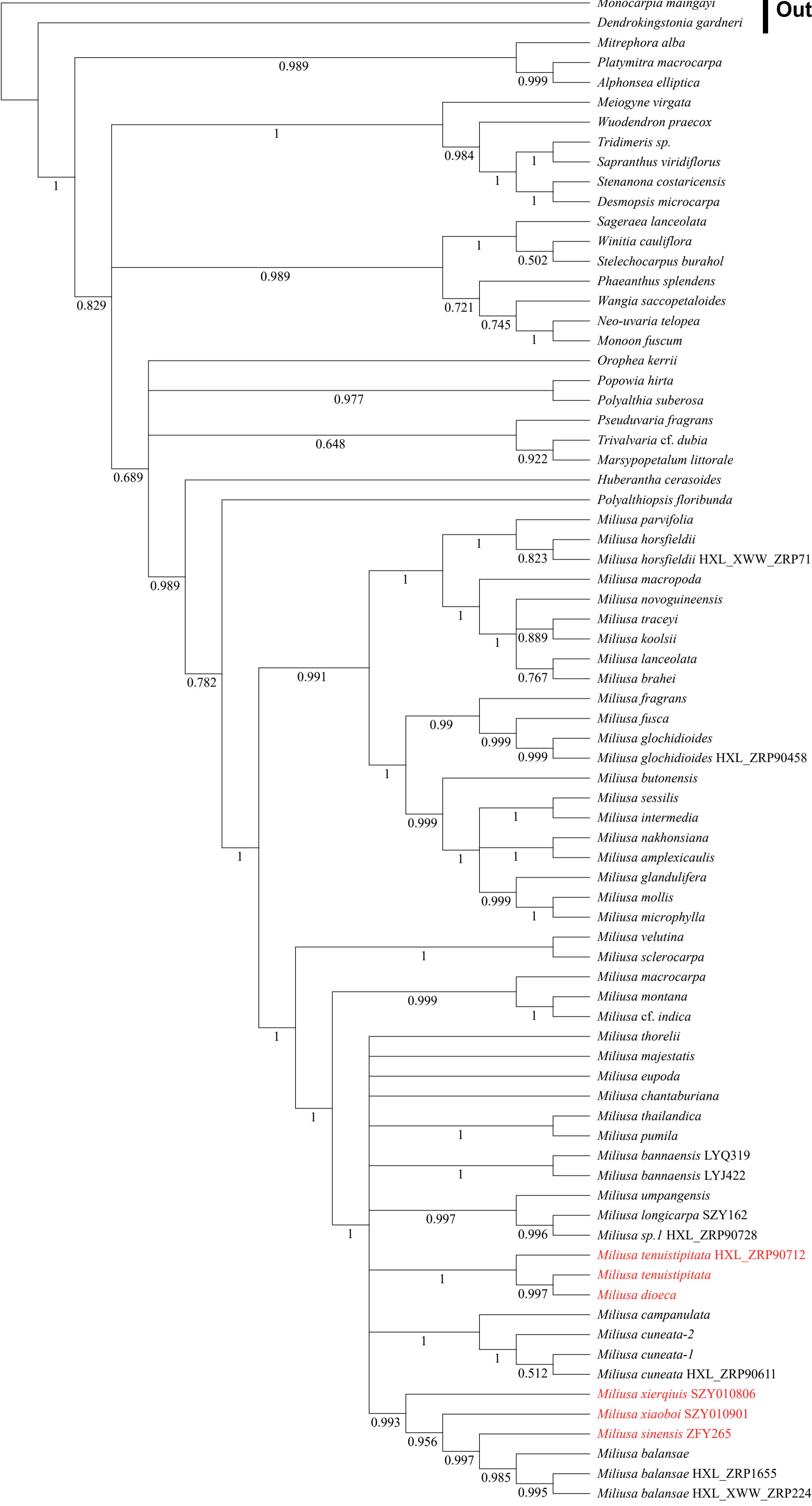

Outgroup

Miliusa

Trib. Miliuseae

Supplement: Supplementary material 4 — Bayesian phylogenetic analysis of the Miliusa in China [file phytokeys-273-185_article-174592__-s004.pdf]
